# Undiagnosed HIV infections among gay and bisexual men increasingly contribute to new infections in Australia

**DOI:** 10.1002/jia2.25104

**Published:** 2018-04-11

**Authors:** Richard T Gray, David P Wilson, Rebecca J Guy, Mark Stoové, Margaret E Hellard, Garrett P Prestage, Toby Lea, John de Wit, Martin Holt

**Affiliations:** ^1^ The Kirby Institute UNSW Sydney Sydney NSW Australia; ^2^ Burnet Institute Melbourne VIC Australia; ^3^ School of Public Health and Preventive Medicine Monash University Melbourne VIC Australia; ^4^ Department of Infectious Diseases The Alfred Hospital Melbourne VIC Australia; ^5^ German Institute for Addiction and Prevention Research (DISuP) Catholic University of Applied Sciences North Rhine‐Westphalia Germany; ^6^ Centre for Social Research in Health UNSW Sydney Sydney NSW Australia

**Keywords:** Australia, gay and bisexual men, mathematical model, transmission dynamics, undiagnosed HIV, HIV cascade

## Abstract

**Introduction:**

We determined the contribution of undiagnosed HIV to new infections among gay and bisexual men (GBM) over a 12‐year period in Australia where there has been increasing focus on improving testing and HIV treatment coverage.

**Methods:**

We generated annual estimates for each step of the HIV cascade and the number of new HIV infections for GBM in Australia over 2004 to 2015 using relevant national data. Using Bayesian melding we then fitted a quantitative model to the cascade and incidence estimates to infer relative transmission coefficients associated with being undiagnosed, diagnosed and not on ART, on ART with unsuppressed virus, or on ART with suppressed virus.

**Results:**

Between 2004 and 2015, we estimated the percentage of GBM with HIV in Australia who were unaware of their status to have decreased from 14.5% to 7.5%. During the same period, there was a substantial increase in the number and proportion of GBM living with HIV on treatment and with suppressed virus, with the number of virally suppressed GBM increasing from around 3900 (30.2% of all GBM living with HIV) in 2004 to around 14,000 (73.7% of all GBM living with HIV) in 2015. Despite the increase in viral suppression, the annual number of new infections rose from around 660 to around 760 over this period. Our results have a wide range due to the uncertainty in the cascade estimates and transmission coefficients. Nevertheless, undiagnosed GBM increasingly appear to contribute to new infections. The proportion of new infections attributable to undiagnosed GBM almost doubled from 33% in 2004 to 59% in 2015. Only a small proportion (<7%) originated from GBM with suppressed virus.

**Discussion:**

Our study suggests that an increase in HIV treatment coverage in Australia has reduced the overall risk of HIV transmission from people living with HIV. However, the proportion of infections and the rate of transmission from undiagnosed GBM has increased substantially. These findings highlight the importance of HIV testing and intensified prevention for Australian GBM at high risk of HIV.

## 1 Introduction

People with undiagnosed HIV contribute disproportionately to HIV transmission because the early stages of infection are the most infectious, and without treatment an individual remains infectious 1, 2, 3, 4, 5, 6, 7. Being unaware of their infection undiagnosed individuals are also less likely to take precautions to prevent transmission 8.In high‐income countries, with concentrated epidemics among gay, bisexual and other men who have sex with men (GBM), the proportion of people living with HIV with undiagnosed infection ranges from 10% to 50% 2, 3, 4, 5, 9, 10, 11, 12 with modelling suggesting the majority of new HIV infections originate from GBM who are undiagnosed or diagnosed but not in care 1, 6, 10, 13.

Reducing undiagnosed HIV and increasing treatment uptake through more frequent testing and timely diagnosis have become critical to global efforts to reducing HIV transmission 14. Many countries monitor their progress towards global targets using a HIV cascade 15, 16. The cascade starts with the number of people living with HIV and shows the number or proportion of people who are diagnosed, retained in care, on treatment and virally suppressed.

In Australian jurisdictions, the proportion of undiagnosed GBM was reported to be 20 to 31% in 2007 to 2008 17, 18 compared to 9% nationally in 2014 19. While there were differences in study methodology and wide confidence intervals for earlier estimates, these studies suggest that the prevalence of undiagnosed HIV has fallen over the last decade. In parallel, there have been increases in HIV testing, substantial increases in HIV treatment coverage and reductions in population viral load 11. Yet during the same period, annual HIV notifications increased in Australia, stabilizing at around 1000 to 1100 infections per year, with 65% to 70% occurring in GBM 11. Similar increases in HIV incidence have been seen in other locations with concentrated epidemics among GBM despite an increase in treatment coverage (like British Columbia as of 2015 20, 21). Whereas in San Francisco, a decline in undiagnosed infection following increases in HIV testing and treatment coverage coincided with a decline in HIV incidence 22, 23, 24.

Many models have been used to estimate the role of undiagnosed infection in sustaining epidemics and assessing the impact of interventions along the treatment cascade. Many of these models are complex static HIV transmission models (providing point estimates) or dynamic HIV transmission models (to provide estimates over time) incorporating risk group, sexual behaviour and disease progression data 1, 3, 6, 13, 25, 26. Such models require a large amount of demographic, behavioural, and clinical data and considerable effort to calibrate and update. An alternative approach, previously developed by Kelly and Wilson 27, uses a simple calculation to estimate the average infectivity of people living with HIV over time. We applied this approach, to estimate the proportion of new HIV infections in Australian GBM attributed to each step of the HIV cascade over time.

## 2 Methods

Using the Australian HIV cascade, we obtained annual estimates for the number of Australian GBM living with HIV who have undiagnosed infection $N_{u}$, have been diagnosed but are not on treatment $N_{d}$, are taking treatment but have an unsuppressed viral load $N_{t}^{u}$, and are on treatment and have a suppressed viral load $N_{t}^{s}$ (Data S1). We then linked estimates for the annual number of new GBM infections *I* to each stage of the cascade, inferring a transmission coefficient, equal to the “average” number of transmissions from people living with HIV in each step of the cascade each year (denoted by $\;\beta_{u}$,$\;\beta_{d}$,$\;\beta_{t}^{u}$, and $\;\beta_{t}^{s}$). Mathematically, this is described by the following equation

(1) $$I\left(t\right)=\beta_{u}\left(t\right)N_{u}\left(t\right)+\beta_{d}\left(t\right)N_{d}\left(t\right)+\beta_{t}^{u}\left(t\right)N_{t}^{u}\left(t\right)+\;\beta_{t}^{s}\left(t\right)N_{t}^{s}\left(t\right)$$

for each point in time *t*. If estimates for the number of new infections and the number of GBM in each step of the HIV cascade are available over time, it is possible to fit the transmission coefficients, which can then be used to estimate the proportion of new infections attributable to each step of the cascade; for example, for undiagnosed this is$\;\beta_{u}N_{u}/I$. This model does not explicitly capture changes in behaviour or clinical factors that cause changes in the transmission coefficients over time. The coefficients simply describe the overall transmission likelihood from people at each step of the cascade.

We now summarise the methods we used to estimate the number of GBM in each step of the cascade in Australia, the number of new infections, and the transmission coefficient fitting methodology (details provided in the Data S1). We conducted this analysis using R version 3.2.2 28. Reproducible code, cleaned input data and summary results are available online 29. No ethical approval or consent was sought as this was a mathematical modelling study using publicly available data.

### 2.1 HIV cascade for Australian gay bisexual men

We estimated four steps of the HIV cascade for Australian GBM using a variation in the methodology described in national surveillance reporting 11. To estimate the proportion undiagnosed, we used the European Centre for Disease Control (ECDC) HIV Modelling Tool 30, 31. The ECDC tool is a multi‐state back‐calculation model that fits diagnoses rates over time using data on new HIV and AIDS diagnoses, estimates for the number of annual deaths and emigrations within people living with diagnosed HIV, and estimates for the rate of CD4 decline. We applied the ECDC model to GBM using HIV surveillance data from the Australian National HIV Registry 11. The calculations for the other steps of the cascade are described in Data S1.

For each step of the cascade, we provide an annual best estimate and range, rounded to the nearest 10. We defined GBM living with HIV who have never been diagnosed in Australia as “undiagnosed”, those previously diagnosed but not on ART as “diagnosed”, those on ART but with viral load ≥200 copies/mL as “unsuppressed”, and those on ART and with viral load <200 copies/mL as “suppressed.”

### 2.2 New infections

**Figure 1:**
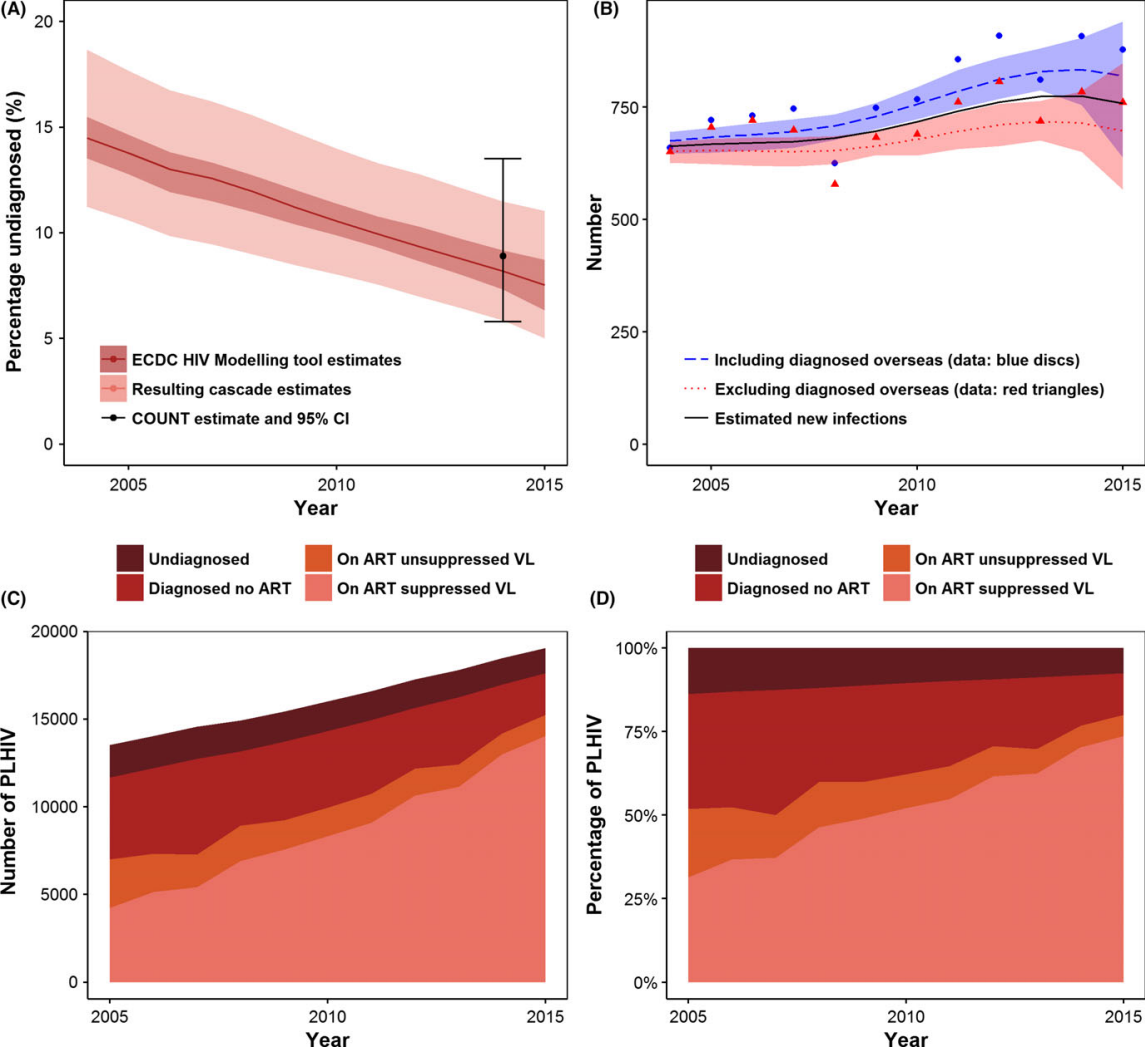
Estimates for new infections and the Australian GBM HIV cascade. **(A)** Estimated percentage of people undiagnosed over 2004 to 2015 compared to the 2014 COUNT estimate. The red line is the average of the two estimates produced by the two ECDC HIV Modelling Tool scenarios. The dark red band is the range in the percentage undiagnosed from the ECDC tool and the lighter band is the overall range in the percentage undiagnosed once the uncertainty in the number diagnosed is included. The black point and error bar show the point estimate and 95% confidence interval for percentage undiagnosed nationally from the COUNT study**. (B)** Estimated number of new infections from the ECDC HIV modelling tool with people previously diagnosed overseas (blue) and with those people excluded (red). The blue and red points correspond to the respective number of notifications attributed to male‐to‐male sex. The black line is the best estimate used for the analysis. The HIV cascade for Australian GBM during 2004 to 2015 with the number **(C)** and proportion **(D)** of GBM living with HIV who are undiagnosed, diagnosed but not on ART, on ART but with detectable (unsuppressed) VL, and on ART with undetectable (suppressed) VL. (Time trends for each population and uncertainty in the estimates are shown in Data S1).

The ECDC tool also produces estimates for new infections. As arrival date for people previously diagnosed overseas is incomplete in the HIV registry, we ran the ECDC tool under two scenarios, once with people previously diagnosed overseas included in the notifications (as used for the undiagnosed estimate) and once with them excluded. We used the midpoint as the best estimate for new infections with the lower bound equalling the lower bound of the scenario with people previously diagnosed excluded and the upper bound equalling the upper bound of the scenario with people previously diagnosed included (Figure 1B). We provide the final ECDC HIV Modelling Tool output spreadsheets in the code repository 29.

### 2.3 Model fitting procedure

We fitted the transmission coefficients to the HIV cascade and new infections estimates using a Bayesian melding methodology 32, 33, 34. This allowed us to incorporate the uncertainty in the cascade and new infection estimates as well as the impact of ART on preventing HIV transmission.

The first step specifies prior probability distributions (priors) for each transmission coefficient. Rather than specifying priors for the number of people in each step of the HIV cascade and each transmission coefficient, we used the point estimates for each cascade step and merged the uncertainty in the cascade values into the transmission coefficients. We did this as follows, letting $N_{x}$ represent the point estimate for each cascade step then the range of values for each step can be written as $N_{x}E_{x}$, where $E_{x}$ represents a multiplicative factor essentially representing the uncertainty in the population estimate for step x. Each term in Equation (1) can then be written as $\beta_{x}E_{x}N_{x}$. As the transmission coefficients $\beta_{x}$ and uncertainty terms $E_{x}$ are multiplied, they can be represented by one overall transmission coefficient $\beta_{x}^{\prime }=\;\beta_{x}E_{x}$ which implicitly includes the uncertainty in both the cascade values and the transmission coefficient. Using the point estimates for each cascade step in this way means we only require priors for each $\beta_{x}^{\prime }$ term. For the remainder of this study we implicitly assume the transmission coefficients $\beta_{x}\;$in Eq. (1). multiply the corresponding point estimate for each cascade step.

We assumed uniform priors for the transmission coefficients for the non‐suppressed steps with a range large enough to capture the plausible values for these parameters (capturing the uncertainty in transmission and the cascade estimates; see Table 1). For the suppressed population we used an exponential distribution with a 95% confidence interval 0 to 0.0084 based on the results from the PARTNER Study 35. Using the specified priors, we applied a sampling‐importance‐resampling algorithm to determine the posterior probability distributions (posteriors) for each transmission coefficient (described in Data S1). We took 5 million samples of the prior distributions and then re‐sampled the resulting set of parameters 100,000 times with probability proportional to the calculated sampling weights to approximate the posterior distribution for each transmission coefficient.

**Table 1:** Specification of model parameter priors

Parameter	Symbol	Prior distribution	Notes
Rate of transmission from GBM with undiagnosed HIV infection to susceptible GBM (per 1000 people)	$\beta_{u}$	Uniform [3.9, 2105]	Assumed broad range based on the number of HIV notifications per person living with HIV as described for $\beta_{d}$ (in the row below) multiplied by a factor reflecting potential changes in sexual behaviour post‐diagnosis and a likely higher viral load during early infection pre‐diagnosis. Generally GBM reduce behaviour associated with HIV transmission 8, 45, 46, however, we assume this factor ranges from 0.9 reflecting the potential for some GBM to increase sexual activity post‐diagnosis, to 30 which reflects higher levels of behaviour contributing to the risk of acquiring HIV 8, 45, 46 and the relative increase in transmissibility due to early infection 40, 47, 48. The resulting upper and lower bounds are then multiplied by the mean relative difference between the point estimate and lower and upper bounds of the range (0.86 to 1.17) for the number of people with undiagnosed infection over 2004 to 2015. Lower = 1000 * 0.005 * 0.9 * 0.86; upper = 1000 * 0.06 * 30 * 1.17.
Rate of transmission from GBM with diagnosed HIV infection not on ART to susceptible GBM (per 1000 people)	$\beta_{d}$	Uniform [3.5, 78]	Assumed range based on the number of HIV notifications per person living with HIV from Australia's Annual Surveillance Report 41. This has ranged between 6 and 4 new diagnoses per 100 people living with diagnosed HIV. Given this includes people on ART and with suppressed virus we assume an upper bound of 6 per 100 people. For the lower bound we assume a small rate (0.5 per 100 people) to reflect the potential reduction in sexual activity initially post diagnosis. These rates were then multiplied by the mean relative difference between the point estimate and lower and upper bounds of the range (0.7 to 1.3) for the number of people diagnosed but not on ART over 2004 to 2015. Lower value = 1000*0.005*0.7; upper value = 1000*0.06 *1.3.
Rate of transmission from GBM on ART but with unsuppressed virus to susceptible GBM (per 1000 people)	$\beta_{t}^{u}$	Uniform [0.08, 203]	Assumed broad range based on the range in the number of HIV notifications per person living with diagnosed HIV as described for $\beta_{d}$ (in the row above) multiplied by a factor reflecting potential changes in sexual behaviour upon the start of ART. We assume this factor ranges from 0.05, reflecting the potential for some GBM to almost stop sexual activity, as they are aware they have not achieved viral suppression, to 2 reflecting a potential increase in sexual behaviour due to a belief that ART prevents transmission. The resulting upper and lower bounds are then multiplied by the mean relative difference between the point estimate and lower and upper range (0.33 to 1.7) for the number of people on ART with unsuppressed virus over 2004 to 2015. Lower value = 1000*0.005*0.05*0.33; upper value = 1000*0.06*2*1.7.
Rate of transmission from GBM on ART but with suppressed virus to susceptible GBM (per 1000 people)	$\beta_{t}^{s}$	Exponential:Mean: 1/2.8	Assumed distribution based on the results of the PARTNER study that reported 0 transmissions due to any sex between serodiscordant men who have sex with men (with the HIV‐positive partner having suppressed virus) in 418 couple‐years of follow‐up 35. Using the exact Poisson method, the study estimated the upper limit of the 95% confidence interval to be 8.4 transmissions per 1000 couple‐years. We used an exponential prior with a mean equal to 2.8 transmissions per 1000 people such that the 95% quantile equals 8.4 transmissions per 1000 people. Note samples from this prior were multiplied by sampled values from a uniform distribution with a range given by the mean relative difference between the point estimate and lower and upper range (0.89 to 1.12) for the number of people on ART with suppressed virus over 2004 to 2015 to capture the uncertainty in the number with suppressed virus.

Prior distributions of model parameters in Equation (1) with justifications. We used the same distribution for separately sampling the transmission coefficient prior in 2004 and in 2015 to produce distinct posterior distributions for the start and end of the analysis period.

We initially applied this procedure assuming constant transmission coefficients. However, we obtained a better fit to new infections by assuming a linear change from a start value in 2004 to an end value in 2015. This is the simplest time varying assumption but is reasonable as relevant behavioural indicators (such as condomless anal intercourse with casual partners, partner numbers and testing rates) have changed gradually and linearly over time 36, 37, 38, 39. For each transmission coefficient, we sampled two priors from the same distribution, one for the start value in 2004, and one for the end value in 2015. We assumed the transmission coefficient related to suppressed virus was constant over time as the current evidence suggests the transmission probability from an infected person with suppressed virus is close to zero 35, 40. This means changes in behaviour (such as a reduction in condom use) will have a minimal effect on transmission from the suppressed population. Data S1 of the Supplementary Material show the priors and the resulting posteriors.

We used the mean or median of the full posterior set to produce all the results with the 2.5th and 97.5th percentiles used to produce 95% credible intervals (95% CrIs). We rounded the final cascade estimates to the nearest whole number.

### 2.4 Sensitivity analysis

To assess the effect of changing the suppressed transmission assumption and the robustness of our methodology we ran four alternative scenarios: (1) using a prior that describes a relative reduction in transmission for the suppressed population compared to the diagnosed population which was informed by the Cohen et al. study in 2011 40; (2) assuming GBM living with HIV with suppressed virus are not infectious; (3) assuming no uncertainty in the HIV cascade estimates for 2004 to 2015; and (4) the methodology for the 2004 to 2014 Australian GBM HIV cascade estimates which assumed a lower overseas migration rate and defined a viral load <400 copies/mL for suppression 41. We describe these scenarios in more detail and provide the complete results of these analyses in Data S1 respectively of the Supplementary Material.

## 3 Results

The percentage of GBM with undiagnosed HIV infection in Australia decreased from 14.5% (range 11.2% to 18.7%) to 7.5% (range 5.0% to 11.0%) during 2004 to 2015 with the number undiagnosed declining slightly (Figure 1A, C, Table 2). The estimated percentage undiagnosed was similar to that observed in a 2014 national prevalence study with a range within the 95% confidence interval of the study (Figure 1A) 19. This fall in the proportion with undiagnosed HIV was mainly due to the 47% increase in the number of GBM living with HIV (i.e. an increasing denominator; Figure 1C, Table 2). Figure 1C shows there was a substantial increase in the number and proportion of GBM living with HIV on treatment and with suppressed virus (see Table 2). Despite the level of viral suppression increasing, the estimated number of new infections rose 14.5% according to our ECDC model estimates (Figure 1B; Data S1) – although the number of new infections per 1000 GBM living with HIV decreased from 49.3 to 36.2 (Table 2). We provide all estimates and ranges for the HIV cascade steps and new infections in Data S1 of the Supplementary Material.

**Table 2:** Cascade estimates, transmission coefficients and new infections attributed to each step of the Australian GBM HIV cascade for 2004, 2010 and 2015

Population	Year	Number of people (mean, range)	Percentage of all people living with HIV (mean, range)	Transmission coefficient (transmission per 1000 people; mean, 95% CrI)	Number of new infections (mean, 95% CrI)	Percentage of all new infections (mean, 95% CrI)
Undiagnosed	2004	1880 (1590 to 2190)	14.5% (11.2% to 18.7%)	110 (8.7 to 280)	212 (16 to 521)	33.2% (2.4% to 80.8%)
	2010	1690 (1420 to 2010)	10.6% (8% to 14%)	–	358 (147 to 559)	49.5% (20.1% to 77.1%)
	2015	1440 (1070 to 1860)	7.5% (5% to 11%)	290 (92 to 470)	423 (133 to 678)	59.1% (20.9% to 89%)
Diagnosed not on ART	2004	4430 (2980 to 5850)	34.1% (21% to 49.8%)	31 (3 to 81)	136 (13 to 360)	21.4% (2% to 57.3%)
	2010	4370 (2070 to 6660)	27.3% (11.7% to 46.3%)	–	165 (40 to 314)	22.7% (5.7% to 43.2%)
	2015	2380 (0 to 5790)	12.5% (0% to 34.3%)	43 (3.7 to 93)	103 (9 to 222)	15% (1.2% to 34.3%)
On ART but unsuppressed	2004	2750 (1720 to 3740)	21.2% (12.2% to 31.8%)	100 (11 to 200)	282 (29 to 562)	43.9% (4.6% to 84.6%)
	2010	1620 (0 to 3500)	10.1% (0% to 24.3%)	–	177 (43 to 334)	24.5% (5.9% to 46.7%)
	2015	1200 (0 to 4410)	6.3% (0 to 26.1%)	110 (5.2 to 260)	138 (6 to 308)	19.7% (0.9 to 45.8%)
On ART and suppressed	2004	3920 (3450 to 4390)	30.2% (24.4 to 37.3%)	2.5 (0.059 to 9)	10 (0 to 35)	1.5% (0 to 5.6%)
	2010	8340 (7390 to 9300)	52.1% (41.8 to 64.7%)	–	24 (3 to 70)	3.3% (0.4 to 9.7%)
	2015	1,4050 (12,440 to 15,740)	73.7% (58.3 to 93.2%)	3.2 (0.086 to 12)	44 (1 to 167)	6.2% (0.2 to 22.5%)
Total	2004	12,980 (11,760 to 14,160)	100%	49.3 (39.3 to 61.6)	640 (556 to 724)	100%
	2010	16,015 (14,380 to 17,680)	100%	–	725 (645 to 805)	100%
	2015	19,070 (16,890 to 21,340)	100%	36.2 (25.3 to 50.8)	690 (539 to 858)	100%

Cascade estimates rounded to nearest 10. Number of new infections rounded to nearest whole number. Transmission coefficients rounded to two significant figures. 95% CrI = 95% credible interval. Only 2004 (start) and 2015 (end) values for the transmission coefficient shown. Results for all years are provided in Data S1 in the Supplementary Material.

**Figure 2:**
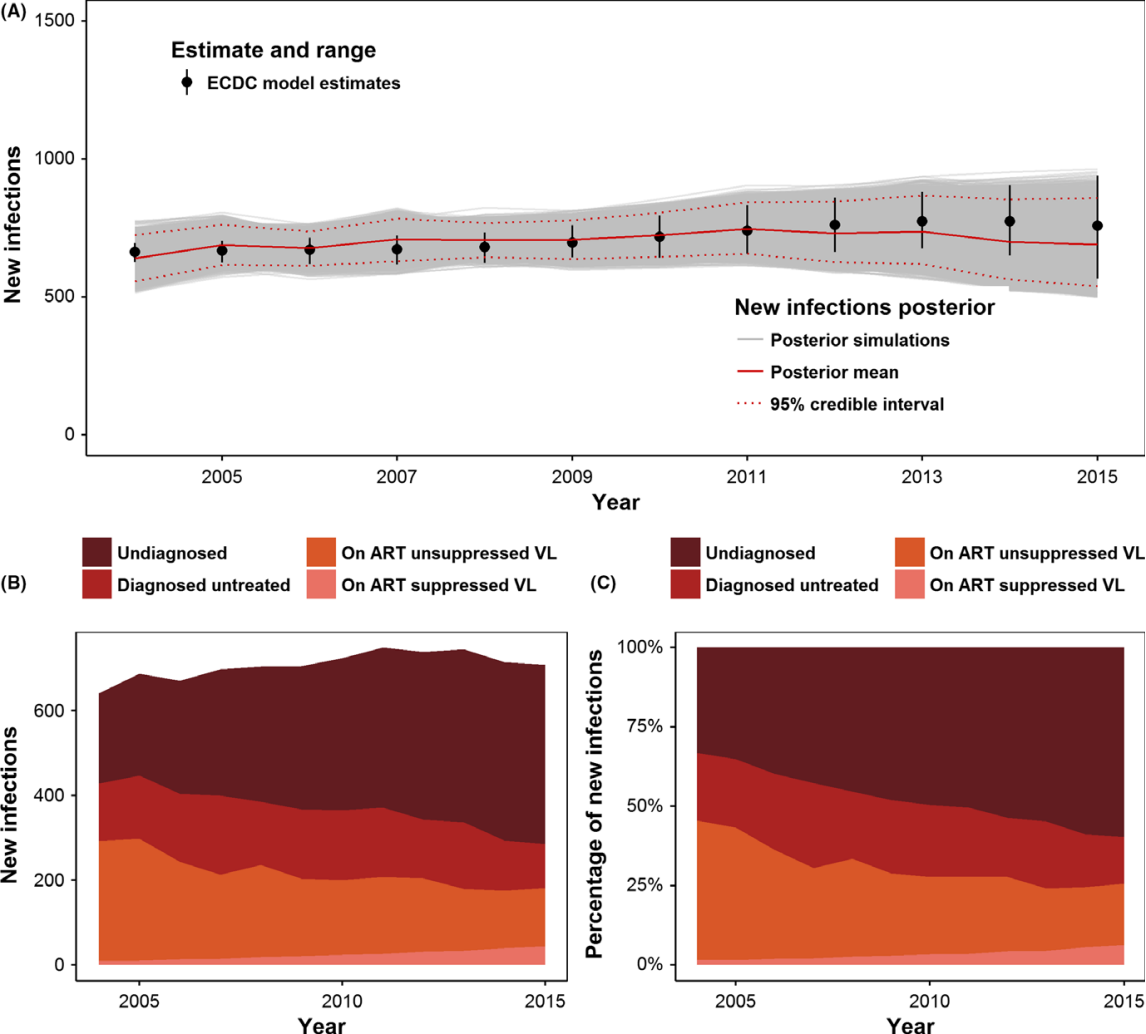
Estimated number and percentage of new infections attributed to each step of the Australian GBM HIV cascade. **(A)** Estimated new infections from the posterior simulations. Each thin grey line is one simulation in the posterior, the thick red line is the posterior mean value at each time point and the dashed red lines are the lower and upper bounds of the 95% credible interval. The black dots and lines show the estimated number new infections and range produced by the ECDC HIV modelling tool. Estimated number **(B)** and proportion **(C)** of overall new infections attributed to each step of the Australia GBM HIV cascade.

The annual posterior mean for the number of new infections closely aligns with the ECDC HIV Modelling Tool estimates during 2004 to 2013 (Figure 2A). The model posterior estimates were below the new infections estimates for 2014 and 2015 but are still well within the 95% confidence intervals and occurred when the ECDC HIV Modelling Tool had the widest uncertainty.

Despite large uncertainty in the estimates (according to the 95% credible intervals), there was a large increase in the number and proportion of infections attributable to undiagnosed infection during 2004 to 2015 (Figure 2B, Table 2), even with a small decline in the number of GBM with undiagnosed infection (see Data S1). The corresponding percentage of new infections attributable to undiagnosed infection increased from 33.2% (95% CrI: 2.4% to 80.8%) in 2004 to 59.1% (95% CrI: 20.9% to 89.0%) in 2015. The number and percentage of new infections attributed to diagnosed and unsuppressed GBM decreased during 2004 to 2015 (Table 2). New infections attributed to GBM on ART with suppressed virus was very small (<7%) but increased during 2004 to 2015 (see Table 2). This is due to the large increase in the number of virally suppressed GBM (Figure 1C) and a small but non‐zero posterior probability of transmission from this group. We provide results from the four alternative assumptions for transmission while virally suppressed in Data S1 of the Supplementary Material.

**Figure 3:**
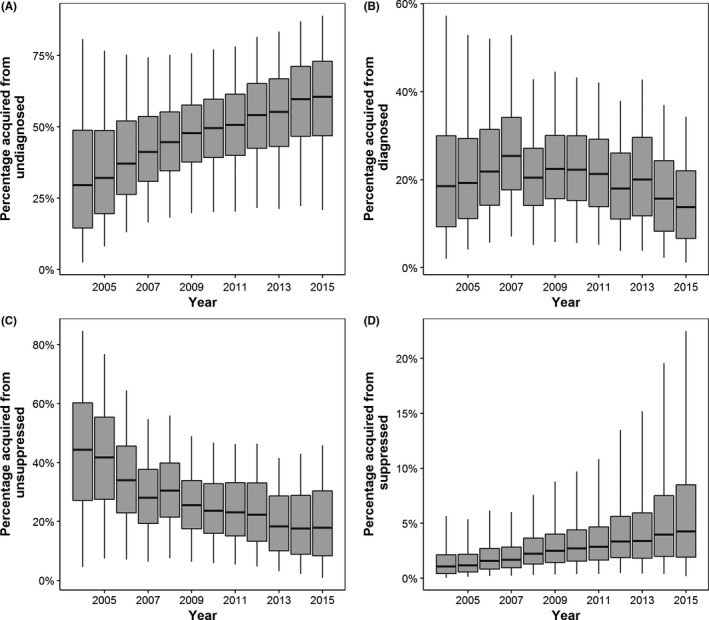
Boxplots of the posterior distribution for percentage of new infections attributed to each step of the Australian GBM HIV cascade for each year during 2004 to 2015: (A) undiagnosed, (B) diagnosed but not on ART, (C) on ART but with detectable (unsuppressed) VL and (D) on ART with undetectable (suppressed) VL. Each box plot shows the median, inter‐quartile range and 95% credible interval of the posterior distributions.

Figure 3 shows the annual posterior distributions (as boxplots) for the percentage of new infections acquired from GBM in each step of the HIV cascade. The resulting posteriors show large uncertainty, reflecting the lower and upper bounds in the cascade estimates and new infections. This uncertainty also reflects our fitting methodology as we fitted seven model parameters using only 12 time points. Except for the suppressed step, this uncertainty generally reduced over time. The posterior peak for the percentage acquired from suppressed GBM remained relatively stable during 2004 to 2015, however, the variance increased resulting in the mean percentage increasing fourfold (see Table 2 and Figure 3).

**Figure 4:**
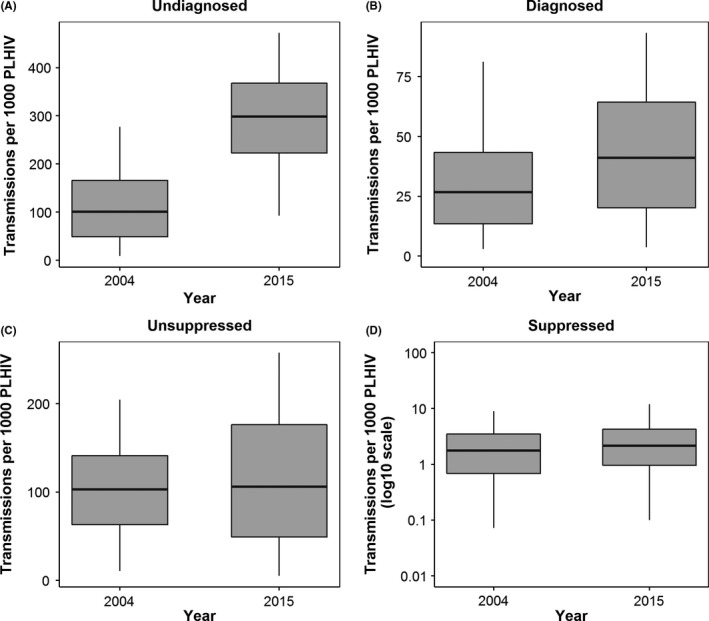
Change in the rate of transmission for people living with HIV in each step of the GBM HIV cascade during 2004 to 2015: (A) undiagnosed, (B) diagnosed but not on ART, (C) on ART but with detectable (unsuppressed) VL and (D) on ART with undetectable (suppressed) VL. Each box plot shows the median, inter‐quartile range and 95% credible interval of the posterior distributions.

Figure 4 shows the change in the rate of transmission per 1000 people living with HIV in each cascade step (reflecting the fitted posterior for each transmission coefficientβ). While the posteriors for each step showed large uncertainty, we estimated a relatively small change in the mean rate of transmission for diagnosed, unsuppressed and suppressed GBM during 2004 to 2015 (39% increase in the mean for diagnosed, 10% increase for unsuppressed and 28% increase for suppressed; see Table 2 and Data S1). In contrast, we estimated a large increase in the rate of transmission from undiagnosed GBM from a mean of 110 (95% CrI: 8.7% to 280) to 290 (95% CrI: 92% to 470) per 1000 undiagnosed GBM during 2004 to 2015, a 2.6‐fold increase over the 12 years. While each transmission coefficient increased during 2004 to 2015 the number of infections per 1000 people living with HIV decreased from 49.3 (95% CrI: 39.3% to 61.6) to 36.2 (95% CrI: 25.3 to 50.8) due to the large increase in the suppressed population. Prior and posterior distributions for the transmission coefficients are in Data S1 of the Supplementary Material.

Our results were relatively robust to changes in the suppression prior and the HIV cascade estimates. Using a suppressed prior based on the relative reduction in transmission compared to the diagnosed step from the Cohen 2011 et al. study 33, the percentage of infections attributed to undiagnosed GBM increased from a mean of 33.3% (95% CrI: 2.2% to 81.3%) in 2004 to a mean of 57.5% (95% CrI: 13.9% to 91.1%) in 2015. This scenario attributed a similar percentage of new infections to suppressed GBM as for the main analysis. When we assumed suppressed GBM were not infectious the percentage of infections attributed to undiagnosed GBM increased more rapidly from a mean of 31.8% (95% CrI: 2.4% to 80.9%) in 2004 to a mean of 65.3% (95% CrI: 28% to 93.1%) in 2015. Assuming a zero range in the estimates for each cascade step resulted in different posteriors for the percentage of infections attributed to undiagnosed GBM with a mean of 47.7% (95% CrI; 13.4% to 83.3%) in 2004 and a mean of 73.6% (95% CrI: 51.3% to 91.7%) in 2015. However, the posterior distributions still overlap the posteriors obtained in our main analysis. Removing the uncertainty in the cascade estimates essentially reduced the variance in the posteriors, as expected, and increased the estimated contribution of undiagnosed to new infections. Finally, applying our methodology to the 2004 to 2014 HIV cascade estimates (which are slightly higher for each step due to a different methodology) only produced a marginally higher estimate for the percentage of new infections from undiagnosed GBM. We provide full details and figures for all the sensitivity results in Data S1 of the Supplementary Material.

## 4 Discussion

We assessed the contribution and role of GBM with undiagnosed HIV to new infections in Australia during 2004 to 2015 using a simple transmission model and Bayesian fitting procedure. Despite a 50% increase in the proportion of GBM living with HIV virally suppressed and the proportion of GBM living with HIV undiagnosed almost halving between 2004 and 2015, the estimated number of new HIV infections rose 8% (consistent with trends in HIV notifications attributed to male‐to‐male sex). It is likely that most new HIV infections among GBM in Australia are now attributable to those who are undiagnosed (59%, 95% CrI: 20.9% to 89.0%), having grown from around a third of infections in 2004. As the proportion attributable to undiagnosed HIV has increased, the number of new infections has increased offsetting the infections prevented through reductions in community viral load.

A large increase in the proportion of infections attributed to undiagnosed GBM is potentially unsurprising given the large increase in treatment and viral suppression among Australian GBM during 2004 to 2015. Such an increase might not necessarily be due to an associated increase in the transmission coefficient for undiagnosed GBM (it is possible, depending on the cascade population sizes, for the proportion of new infections attributed to undiagnosed GBM to increase while the average rate of transmission from this population decreases). The large increase in the transmission coefficient for undiagnosed GBM seen in our analysis could be due to several reasons. First, the overall population of GBM living with HIV has increased substantially since 2004 in Australia and people living with HIV are now significantly more likely to be receiving effective treatment and much less likely to be infectious. Second, condomless sex among GBM in Australia has become more common over the last decade 36, 37, so men with undiagnosed HIV are more likely to engage in sexual practices that facilitate transmission. There has also been an increase in the use of risk reduction practices relying on an accurate knowledge of serostatus by HIV‐negative men, such as serosorting 37, 42. If undiagnosed GBM believe they are HIV‐negative and engage in serosorting or other risk reduction strategies (and do not use condoms), then sexual transmission is more likely to occur. Third, there has been a reduction in time between HIV infection and diagnosis during 2004 to 2015 as HIV testing frequency among GBM has increased 38, 39, with the ECDC model estimating a decline from a mean time of 2.8 years to 1.9 years between infection and diagnosis. At a population level this means a larger proportion of undiagnosed GBM will be in the acute phase of infection and the average viral load (and resulting infectiousness) of undiagnosed GBM will likely have increased during 2004 to 2015 43.

An advantage of our approach is it uses annually updated surveillance data and cascade estimates and is based on a simple transmission model. Our methodology does not require detailed demographic, behavioural and clinical data and can quickly produce estimates. It is also flexible, being able to incorporate changed assumptions, priors or alternative input. The disadvantage of this simplicity is that we cannot understand the potential casual factors affecting temporal changes across the cascade steps. Our estimates also have several limitations. The number and proportion of infections attributed to each step of the cascade have a large amount of uncertainty due to the range in cascade estimates, the use of wide uninformative priors, and because we are fitting 7 parameters with only 12 data points. If better cascade estimates for each step of the GBM cascade were available with narrower priors, then better estimates with smaller ranges would be obtained. We also assumed a linear change in transmission coefficients over time. This assumption appears to be reasonable, as changes in behaviour, such as the increase in condomless sex 36, 37 and changes in HIV testing frequency among GBM have changed relatively linearly over time, and is likely robust over short periods. However, this assumption may be stretched over the 12‐year period used in this analysis as indicated by the poorer, but still acceptable, fit to new infections for 2014 to 2015. The flexibility of our methodology means we could use different assumptions for the change in the rate of transmission for each step of the cascade over time; however, the number of parameters would need to be minimal to prevent over‐parameterization.

## 5 Conclusions

Our study suggests most new infections in Australia are now due to transmissions from undiagnosed GBM. This concentration of new infections from undiagnosed GBM reflects the success of interventions aimed at increasing HIV treatment coverage in the growing population of GBM with diagnosed HIV. However, the rise in notifications during 2004 to 2015 highlights the need to do more to reduce new infections. Our results suggest the need for a renewed focus on interventions for HIV‐negative GBM while maintaining treatment scale‐up and retention in care for people already living with HIV. The increasing contribution of undiagnosed GBM to new infections highlights the importance of HIV testing and the role of novel diagnostic services (such rapid and self‐testing) to reduce the time between infection and diagnosis 44. It also highlights the need for improved prevention methods for GBM at high risk of infection.

## Competing interests

The authors have no competing interests to declare.

## Authors' Contributions

RTG developed the model and performed the modelling analyses with support from DPW. RTG wrote the modelling code and setup the online code repository. RTG took primary responsibility for drafting and redrafting the manuscript, with support from MH, DPW and RG. All authors read, commented on and approved the final manuscript.

## Funding

This analysis was conducted with support from the COUNT study funded by a National Health and Medical Research Council Project Grant (APP1044749), another National Health and Medical Research Council Project Grant (APP1050874) and the Australian Government Department of Health.

The Kirby Institute is funded by the Australian Government Department of Health, and is affiliated with the Faculty of Medicine, UNSW Sydney, Australia. The Centre for Social Research in Health is funded by the Australian Government Department of Health and is part of the Faculty of Arts and Social Sciences, UNSW Sydney, Australia. The Burnet Institute gratefully acknowledges the contribution to this work of the Victorian Operational Infrastructure Support Programme.

The content of this publication is solely the responsibility of the authors and does not necessarily represent the official views of any of the institutions mentioned previously.

## Supporting information


**Data S1.** Methodological details.

## References

[1] Punyacharoensin N , Edmunds WJ , De Angelis D , Delpech V , Hart G , Elford J , et al. Modelling the HIV epidemic among MSM in the United Kingdom: quantifying the contributions to HIV transmission to better inform prevention initiatives. AIDS. 2015;29:339–49. 25686682 10.1097/QAD.0000000000000525

[2] Hamers FF , Phillips AN . Diagnosed and undiagnosed HIV‐infected populations in Europe. HIV Med. 2008;9 Suppl 2 :6–12. 10.1111/j.1468-1293.2008.00584.x 18557863

[3] Marks G , Crepaz N , Janssen RS . Estimating sexual transmission of HIV from persons aware and unaware that they are infected with the virus in the USA. AIDS. 2006;20:1447–50. 16791020 10.1097/01.aids.0000233579.79714.8d

[4] Supervie V , Ndawinz JD , Lodi S , Costagliola D . The undiagnosed HIV epidemic in France and its implications for HIV screening strategies. AIDS. 2014;28:1797. 24681416 10.1097/QAD.0000000000000270 PMC4262966

[5] Gardner EM , McLees MP , Steiner JF , del Rio C , Burman WJ . The spectrum of engagement in HIV care and its relevance to test‐and‐treat strategies for prevention of HIV infection. Clin Infect Dis. 2011;52:793–800. 21367734 10.1093/cid/ciq243 PMC3106261

[6] Phillips AN , Cambiano V , Nakagawa F , Brown AE , Lampe F , Rodger A , et al. Increased HIV incidence in men who have sex with men despite high levels of ART‐induced viral suppression: analysis of an extensively documented epidemic. PLoS ONE. 2013;8:e55312. 23457467 10.1371/journal.pone.0055312 PMC3574102

[7] Wilson DP , Hoare A , Regan DG , Law MG . Importance of promoting HIV testing for preventing secondary transmissions: modelling the Australian HIV epidemic among men who have sex with men. Sex Health. 2009;6:19–33. 19254488 10.1071/sh08081

[8] Fox J , White PJ , Macdonald N , Weber J , McClure M , Fidler S , et al. Reductions in HIV transmission risk behaviour following diagnosis of primary HIV infection: a cohort of high‐risk men who have sex with men. HIV Med. 2009;10:432–8. 19459996 10.1111/j.1468-1293.2009.00708.x

[9] Public Health Agency of Canada . Summary: estimates of HIV incidence, prvalence and proportion undiagnosed in Canada, 2014. Ottawa: Public Health Agency of Canada, Ottawa, Canada; 2015.

[10] Burns DN , DeGruttola V , Pilcher CD , Kretzschmar M , Gordon CM , Flanagan EH , et al. Toward an endgame: finding and engaging people unaware of their HIV‐1 infection in treatment and prevention. AIDS Res Hum Retroviruses. 2014;30:217–24. 24410300 10.1089/aid.2013.0274 PMC3938938

[11] The Kirby Institute . HIV, viral hepatitis and sexually transmissible infections in Australia Annual Surveillance Report 2016. UNSW Sydney, Sydney, Australia: The Kirby Institute; 2016.

[12] Mammone A , Pezzotti P , Regine V , Camoni L , Puro V , Ippolito G , et al. How many people are living with undiagnosed HIV infection? An estimate for Italy, based on surveillance data. AIDS Lond Engl. 2016;30:1131–6. 10.1097/QAD.0000000000001034 PMC4819953 26807973

[13] Skarbinski J , Rosenberg E , Paz‐Bailey G , Hall HI , Rose CE , Viall AH , et al. Human immunodeficiency virus transmission at each step of the care continuum in the United States. JAMA Intern Med. 2015;175:588–96. 25706928 10.1001/jamainternmed.2014.8180

[14] UNAIDS . Ten targets: 2011 United Nations Political Declaration on HIV and AIDS, global progress and lessons learned, 2011–2015. Geneva, Switzerland: UNIADS; 2015.

[15] Nosyk B , Montaner JS , Colley G , Lima VD , Chan K , Heath K , et al. The cascade of HIV care in British Columbia, Canada, 1996–2011: a population‐based retrospective cohort study. Lancet Infect Dis. 2014;14:40–9. 24076277 10.1016/S1473-3099(13)70254-8 PMC4017913

[16] Hull MW , Wu Z , Montaner JS . Optimizing the engagement of care cascade: a critical step to maximize the impact of HIV treatment as prevention. Curr Opin HIV AIDS. 2012;7:579–86. 23076123 10.1097/COH.0b013e3283590617

[17] Pedrana AE , Hellard ME , Wilson K , Guy R , Stoové M . High rates of undiagnosed HIV infections in a community sample of gay men in Melbourne, Australia. J Acquir Immune Defic Syndr. 1999;2012(59):94–9. 10.1097/QAI.0b013e3182396869 21992925

[18] Birrell F , Staunton S , Debattista J , Roudenko N , Rutkin W , Davis C . Pilot of non‐invasive (oral fluid) testing for HIV within a community setting. Sex Health. 2010;7:11–6. 20152090 10.1071/SH09029

[19] Holt M , Lea T , Asselin J , Prestage G , Wilson D , de Wit J , et al. The prevalence and correlates of undiagnosed HIV among Australian gay and bisexual men: results of a national, community‐based, bio‐behavioural survey. J Int AIDS Soc. 2015;18:20526. 26563846 10.7448/IAS.18.1.20526 PMC4643166

[20] BC Centre for Disease Control . HIV in British Columbia: annual surveillance report 2015. Vancouver, Canada: BC Centre for Disease Control; 2017.

[21] Nosyk B , Zang X , Min JE , Krebs E , Lima VD , Milloy M‐J , et al. Relative effects of antiretroviral therapy and harm reduction initiatives on HIV incidence in British Columbia, Canada, 1996–2013: a modelling study. Lancet HIV. 2017;4:e303–10. 28366707 10.1016/S2352-3018(17)30045-0 PMC5494273

[22] Raymond HF , Chen Y‐H , Ick T , Scheer S , Bernstein K , Liska S , et al. A new trend in the HIV epidemic among men who have sex with men, San Francisco, 2004–2011. J Acquir Immune Defic Syndr. 2013;62:584–9. 23334505 10.1097/QAI.0b013e318285febf

[23] Centers for Disease Control and Prevention . HIV surveillance report. Atlanta, USA: Centers for Disease Control and Prevention; 2015.

[24] Hall HI , An Q , Tang T , Song R , Chen M , Green T , et al. Prevalence of diagnosed and undiagnosed HIV infection: United States, 2008–2012. Morb Mortal Wkly Rep. 2015;64:657–62. PMC4584741 26110835

[25] Shah M , Risher K , Berry SA , Dowdy DW . The epidemiologic and economic impact of improving HIV testing, linkage, and retention in care in the United States. Clin Infect Dis 2015;(2)220–9. 26362321 10.1093/cid/civ801 PMC4690480

[26] Shah M , Perry A , Risher K , Kapoor S , Grey J , Sharma A , et al. Effect of the US National HIV/AIDS strategy targets for improved HIV care engagement: a modelling study. Lancet HIV. 2016;3:e140–6. 26939737 10.1016/S2352-3018(16)00007-2 PMC4787987

[27] Kelly SL , Wilson DP . HIV cascade monitoring and simple modeling reveal potential for reductions in HIV incidence. J AcquirImmune Defic Syndr. 2015;69:257–63. 10.1097/QAI.0000000000000655 25886932

[28] R Core Team . R: A language and environment for statistical computing. Vienna, Austria: R Foundation for Statistical Computing 2016 Available from: https://www.R-project.org/

[29] Gray RT . leftygray/Cascade_Incidence: Version corresponding to final manuscript. (Version v1.1‐final). Zenodo. 2017 [https://doi.org/10.5281/zenodo.1117423]. Also available from: https://github.com/leftygray/Cascade_Incidence [cited 2018 Mar 20].

[30] van Sighem A , Nakagawa F , De Angelis D , Quinten C , Bezemer D , de Coul EO , et al. Estimating HIV incidence, time to diagnosis, and the undiagnosed HIV epidemic using routine surveillance data. Epidemiology. 2015;26:653–60. 26214334 10.1097/EDE.0000000000000324 PMC4521901

[31] ECDC HIV modelling tool [software application]. Version 1.2.2. European Centre for Disease Prevention and Control, Stockholm, Sweden, 2016. Available from: https://ecdc.europa.eu/en/publications-data/hiv-modelling-tool [cited 2018 Mar 20]

[32] Hallett TB , Gregson S , Mugurungi O , Gonese E , Garnett GP . Assessing evidence for behaviour change affecting the course of HIV epidemics: a new mathematical modelling approach and application to data from Zimbabwe. Epidemics. 2009;1:108–17. 21352758 10.1016/j.epidem.2009.03.001

[33] Alkema L , Raftery AE , Brown T . Bayesian melding for estimating uncertainty in national HIV prevalence estimates. Sex Trans Infect. 2008;84(Suppl 1):i11–6. 10.1136/sti.2008.029991 PMC2569139 18647860

[34] Poole D , Raftery AE . Inference for deterministic simulation models: the bayesian melding approach. J Am Stat Assoc. 2000;95:1244.

[35] Rodger AJ , Cambiano V , Bruun T , Vernazza P , Collins S , Van Lunzen J , et al. Sexual activity without condoms and risk of HIV transmission in serodifferent couples when the HIV‐positive partner is using suppressive antiretroviral therapy. JAMA. 2016;316:171–81. 27404185 10.1001/jama.2016.5148

[36] Mao L, Adam PTC, de Wit J (Eds). HIV/AIDS, hepatitis and sexually transmissible infections in Australia: annual report of trends in behaviour 2016. Sydney, Australia: Centre for Social Research in Health, UNSW Sydney; 2016.

[37] Holt M , Lea T , Mao L , Zablotska I , Lee E , Hull P , et al. Adapting behavioural surveillance to antiretroviral‐based HIV prevention: reviewing and anticipating trends in the Australian Gay Community Periodic Surveys. Sex Health. 2017;14:72–9. 27567489 10.1071/SH16072

[38] Wilkinson AL , El‐Hayek C , Spelman T , Fairley CK , Leslie D , McBryde ES , et al. A “test and treat” prevention strategy in Australia requires innovative HIV testing models: a cohort study of repeat testing among high‐risk men who have sex with men. Sex Transm Infect. 2016;92:464–6. 26801226 10.1136/sextrans-2015-052421

[39] Wilkinson AL , El‐Hayek C , Spelman T , Fairley C , Leslie D , McBryde E , et al. “Seek, Test, Treat” lessons from Australia: a study of HIV testing patterns from a cohort of men who have sex with men. J Acquir Immune Defic Syndr. 2015;69:460–5. 25835608 10.1097/QAI.0000000000000613

[40] Cohen MS , Chen YQ , McCauley M , Gamble T , Hosseinipour MC , Kumarasamy N , et al. Prevention of HIV‐1 infection with early antiretroviral therapy. N Engl J Med. 2011;365:493–505. 21767103 10.1056/NEJMoa1105243 PMC3200068

[41] The Kirby Institute . HIV, viral hepatitis and sexually transmissible infections in Australia Annual Surveillance Report 2015. UNSW Sydney, Sydney, Australia: The Kirby Institute; 2015.

[42] Mao L , Kippax SC , Holt M , Prestage GP , Zablotska IB , de Wit JB . Rates of condom and non‐condom‐based anal intercourse practices among homosexually active men in Australia: deliberate HIV risk reduction? Sex Transm Infect. 2011;87:489–93. 21764890 10.1136/sextrans-2011-050041

[43] Hollingsworth TD , Anderson RM , Fraser C . HIV‐1 transmission, by stage of infection. J Infect Dis. 2008;198:687–93. 18662132 10.1086/590501

[44] Guy R , Prestage G , Grulich A , Holt M , Conway D , Jamil M , et al. Potential public health benefits of HIV testing occurring at home in Australia. Med J Aust. 2015;202:529–31. 26021364 10.5694/mja14.01210

[45] Gorbach PM , Drumright LN , Daar ES , Little SJ . Transmission behaviors of recently HIV‐infected men who have sex with men. J Acquir Immune Defic Syndr. 2006;1999(42):80–5. 10.1097/01.qai.0000196665.78497.f1 16763494

[46] Marks G , Crepaz N , Senterfitt JW , Janssen RS . Meta‐analysis of high‐risk sexual behavior in persons aware and unaware they are infected with HIV in the United States: implications for HIV prevention programs. J AcquirImmune Defic Syndr. 2005;1999(39):446–53. 10.1097/01.qai.0000151079.33935.79 16010168

[47] Eaton JW , Hallett TB . Why the proportion of transmission during early‐stage HIV infection does not predict the long‐term impact of treatment on HIV incidence. Proc Natl Acad Sci. 2014;111:16202–7. 25313068 10.1073/pnas.1323007111 PMC4234601

[48] Pilcher CD , Tien HC , Eron JJ , Vernazza PL , Leu S‐Y , Stewart PW , et al. Brief but efficient: acute HIV infection and the sexual transmission of HIV. J Infect Dis. 2004;189:1785–92. 15122514 10.1086/386333

