# Supplementary material for: Undiagnosed HIV infections among gay and bisexual men increasingly contribute to new infections in Australia
Source: J Int AIDS Soc. 2018 Apr 11;21(4):e25104. doi: 10.1002/jia2.25104 (PMC5894250; doi:10.1002/jia2.25104)
Supplement: Supplementary file 1 — Data S1. Methodological details. [file JIA2-21-e25104-s001.docx]

**Undiagnosed HIV infections among gay and bisexual men InCREASINGLY contributE to new infections in Australia**

**Supplementary Material**

**Authors:** Richard T. Gray^§1^, David P. Wilson^2^, Rebecca Guy^1^, Mark Stoove^2^, Margaret Hellard^2^, Garrett Prestage^1^, Toby Lea^3^, John de Wit^3^, Martin Holt^3^

1. The Kirby Institute, UNSW Sydney, Sydney NSW 2052, Australia
2. Burnet Institute, Melbourne VIC 3004, Australia
3. Centre for Social Research in Health, The University of New South Wales, Sydney NSW 2052, Australia

**Contents**

[1. Methodological Details 30](#_Toc501377164)

[1.1 GBM HIV Diagnosis and Care Cascade Calculations 30](#_Toc501377165)

[1.2 Model Fitting Algorithm 32](#_Toc501377166)

[1.3 Sensitivity Analysis 33](#_Toc501377167)

[2. Additional Results 35](#_Toc501377168)

[2.1 Estimates for each step of the 2004-2015 cascade and new infections 35](#_Toc501377169)

[2.2 Annual new infections attributable to each step of the Australian GBM HIV cascade 37](#_Toc501377170)

[2.3 Transmission Coefficient Priors and Posteriors for Main Results 39](#_Toc501377171)

[2.4 transmission Coefficient estimates for main results 43](#_Toc501377172)

[3. Sensitivity Analysis results 43](#_Toc501377173)

[3.1 Australian GBM HIV cascade during 2004-2015 with Cohen 2011 suppressed transmision coefficint prior 45](#_Toc501377174)

[3.2 Australian GBM HIV cascade During 2004-2015 with zero suppressed transmission 49](#_Toc501377175)

[3.3 Australian GBM HIV cascade During 2004-2015 with Partner study Suppressed Transmission coefficient and no range in cascade estimates 54](#_Toc501377176)

[3.4 Australian GBM HIV cascade During 2004-2014 with Cohen 2011 suppressed transmission coefficient prior and the 2014 HIV cascade methodology 58](#_Toc501377177)

[Estimates for each step of the 2004-2014 cascade and new infections 59](#_Toc501377178)

[Results 62](#_Toc501377179)

[4. References 67](#_Toc501377180)

This supplementary material provides further methodological details, additional results related to the results presented in the main paper, and the results of the sensitivity analysis.

1. **Methodological Details**

The following sections provide additional methodological details for the HIV cascade calculations, the Bayesian melding algorithm, and the sensitivity analysis.

- 1. **GBM HIV Diagnosis and Care Cascade Calculations**

We estimated four steps of the HIV cascade for Australian GBM using a variation of the methodology described in national surveillance reporting [1]. The four steps we considered Supplementary Table S1 provides a description and definition of the cascade steps we estimate.

**Supplementary Table S1:** **Definitions for the steps of the Australian GBM HIV cascade.**

| **Cascade Step** | **Definition** |
| --- | --- |
| Number of people living with HIV | Estimated number of people living with HIV in the overall population who were resident in Australia in last 12 months |
| Number of people living with diagnosed HIV | Estimated number of people living with HIV who were resident in Australia in last 12 months who have been diagnosed with HIV |
| Number of people living with diagnosed HIV taking antiretroviral treatment (ART) | Number of people living with diagnosed HIV who received antiretroviral treatment during last 12 months |
| Number of people living with diagnosed HIV on ART with suppressed virus | Number of people living with diagnosed HIV taking ART in the last 12 months who had a viral load (VL) less than 200 HIV-1 RNA copies/ml at last test |

**Number of GBM living with diagnosed HIV**

We first estimated the annual number of GBM living with diagnosed infection by cumulatively adding the number of notifications attributed to transmission through male-to-male sex from Australia’s National HIV Registry, adjusting for duplicates, removing deaths and the number of people who have migrated overseas. A detailed description of the data sources and calculation methodology is available in the 2015 Annual Surveillance Report [1] (with code available online [2]).

Note that people are only reported to Australia’s National HIV registry when they are diagnosed within Australia. This means people who migrate to Australia after they have been diagnosed overseas are not treated as diagnosed in the cascade until they are re-diagnosed in Australia (and are hence classified as undiagnosed until then). We therefore did not consider in-migration of GBM living with HIV.

**Number of GBM living with undiagnosed HIV**

We estimated the proportion of GBM living with HIV who have never been diagnosed in Australia using the European Centre for Disease Control (ECDC) HIV Modelling Tool (available from: <http://ecdc.europa.eu/en/healthtopics/aids/Pages/hiv-modelling-tool.aspx>) [3,4] The ECDC tool is a multi-state back-calculation model using surveillance data on new HIV and AIDS diagnoses, estimates for the number of annual deaths and emigrations within people living with diagnosed HIV, and estimates for the rate of CD4 decline to fit diagnoses rates over time. The ECDC model tool has been used in other countries and detailed description of the methods used by the model are provided elsewhere [3,4]. It produces estimates and ranges for the number of new HIV infections, the number of people living with HIV, mean time from infection to diagnosis, and the number and percentage undiagnosed. The ECDC tool primary uses estimates for the number of annual HIV and AIDS diagnoses and the recorded CD4 count at diagnosis. The model adjusts for missing CD4 count at diagnosis data. Using the resulting undiagnosed proportion, we estimated the overall number of GBM living with HIV in Australia and the number undiagnosed.

The ECDC model includes two methods for producing estimates. We used the incidence method [3], which requires estimates for annual HIV, AIDS, and HIV/AIDS notifications as well as the number of deaths and the number who move overseas post diagnosis. Further, the overall number of annual notifications is broken down into four stages of CD4 count at diagnosis: > 500 cells/µL, 350-500 cells /µL, 200-349 cells/µL and < 200 cells/µL. Given we assumed people previously diagnosed overseas are undiagnosed until they are re-diagnosed in Australia, we ran the ECDC model with people previously diagnosed overseas included in the notifications. Annual notifications were adjusted to reflect duplications, deaths and overseas migration for each step.

We adjusted the ECDC model testing rate parameters (by CD4 count and period) to best fit the data of CD4 count at diagnosis. The ECDC model was run 100 times to produce best estimates and 95% confidence intervals for the results. We used confidence intervals for the ranges in this analysis. The output spreadsheets produced by the ECDC model are available in the online repository [5].

**Number on treatment and with suppressed virus**

We estimated the number of GBM on ART by multiplying the number of people living with diagnosed HIV by the proportion on treatment reported in behavioural surveillance [1,6,7]. While these proportions are based on self-reporting, they align with estimates from enhanced sentinel surveillance of laboratories and clinics [1]. The resulting number on treatment with suppressed virus was then estimated from GBM participating in the Australian HIV Observational Database (AHOD) for 2004-2015 using the proportion of people on treatment with a viral load at last test < 200 HIV-1 RNA copies/ml [1]. Again, these proportions align with other sources [1]. We used the corresponding 95% confidence interval as the range.

- 1. **Model Fitting Algorithm**

As summarised in the main text we fitted a model linking the total number of annual infections in GBM to the number of GBM living with HIV in each stage of the HIV cascade. Mathematically this model is described by

$\begin{matrix} I(t)=\beta_{u}{\left( t \right)N}_{u}(t)+\beta_{d}{\left( t \right)N}_{d}(t)+\beta_{t}^{u}(t)N_{t}^{u}(t)+ \beta_{t}^{s}(t)N_{t}^{s}(t) & (1) \end{matrix}$,

for each point in time $t$ where:

- $N_{u}$ is the number of Australian GBM living with HIV who have undiagnosed infection
- $N_{d}$ is the number of Australian GBM living with HIV who have been diagnosed but are not on treatment
- $N_{t}^{u}$ is the number of Australian GBM living with HIV who have been diagnosed are taking treatment but have an unsuppressed viral load
- $N_{t}^{s}$ is the number of Australian GBM living with HIV who have been diagnosed who have been diagnosed are on treatment but have a suppressed viral load
- $I$ is the estimated number of new GBM infections $I$
- $\beta_{u}$is the average annual number of transmissions from undiagnosed GBM
- $\beta_{d}$ is the average annual number of transmissions from diagnosed GBM
- $\beta_{t}^{u}$ is the average annual number of transmissions from unsuppressed GBM
- $\beta_{t}^{s}$ is the average annual number of transmissions suppressed GBM

We describe the parameters$\beta_{u}$,$\beta_{d}$,$\beta_{t}^{u}$ and $\beta_{t}^{s}$ as transmission coefficients. Using our estimates for the number of new infections $I$ and the number of GBM in each step of the HIV cascade ($N_{u}$, $N_{d}$, $N_{t}^{u}$, and $N_{t}^{s}$over time we can then fit equation to estimate the values of each transmission coefficient and estimate the contribution of each step of the HIV cascade to new infections.

To fit the transmission coefficients in Eq. 1 we used a Bayesian melding methodology [8–10]. This approach consists of the following procedure:

1. Specify prior distributions for each transmission coefficient$\beta$.
2. Generate a set of parameters by randomly sampling from the prior distributions for each transmission coefficient $\beta$.
3. Calculate the estimated number of new infections for this set of parameters using Eq. 1
4. Calculate the sampling weight for the parameter set using the product of the likelihood (probability of obtaining the new infections given the parameter values; described below) and the direct prior (given by the product of the individual parameter priors).
5. Repeat steps 2-4, $n$ times.
6. Resample from the generated ensemble of parameter sets $m$ times, with probability proportional to the sampling weights, to approximate the posterior distributions for the inputs.

To calculate the sampling weight of each parameter set, we assumed a normally distributed likelihood for the difference between the new infections data and the resulting model output. For calculation purposes, we calculated the weight as:

$W= \prod_{t} N\left( d_{t} \right|\mu=0, \sigma=\sigma_{t}) /N(0| \mu=0, \sigma=\sigma_{t})$,

where $d_{t}$ is the difference between the new infections data at time $t$ and the model output for the given parameter set, $\sigma_{t}$ is the estimated standard deviation for the new infections data at time $t$, and $N$ is a normal distribution with mean $\mu$ and standard deviation $\sigma$.

- 1. **Sensitivity Analysis**

As described in the main text, for our results we used estimates for the Australian GBM HIV cascade over 2004-2015 and a prior for the transmission coefficient of suppressed GBM based on the PARTNER study [11]. To assess the effect of changing this suppressed transmission assumption and the robustness of our methodology to changes in HIV cascade estimates we applied our methodology to four alternative scenarios.

We based these scenarios on combinations of the following changes to the suppressed GBM transmission coefficient prior and HIV cascade estimates:

1. We explored changes in the suppressed transmission prior by modifying Eq. 1 to include a term$f_{t}^{s}$, which describes the relative reduction in transmission probability relative to people in the diagnosed but not on treatment step,

$\begin{matrix} I(t)=\beta_{u}{\left( t \right)N}_{u}(t)+\beta_{d}{\left( t \right)N}_{d}(t)+\beta_{t}^{u}(t)N_{t}^{u}(t)+ f_{t}^{s}\beta_{d}(t)N_{t}^{s}(t) & (2) \end{matrix}.$

We specified a prior for this relative reduction in the suppressed transmission coefficient compared to the transmission coefficient for GBM diagnosed but not on ART based on the results from the Cohen et al study in 2011—with a prior given by a lognormal distribution with mean 0.04 (95% CI: 0.01-0.27) [12].

1. Assumed GBM living with HIV with suppressed virus are not infectious; i.e, $\beta_{t}^{s}(t)$ in Eq. 1 equals zero.
2. Assumed no uncertainty in the HIV cascade estimates for 2004-2015
3. Used the 2014 GBM HIV cascade methodology for the 2004-2014 Australian GBM HIV cascade estimates which assumed a lower overseas migration rate and defined a viral load < 400 copies/ml for suppression [13].

1. **Additional Results**

The following sections provide additional figures and tables for the results presented in the main text.

- 1. **Estimates for each step of the 2004-2015 cascade and new infections**

**Supplementary Table S2.** **Australian GBM HIV cascade estimates and ECDC estimates of new infections for 2004-2015.**

|  | Undiagnosed | | Diagnosed | | Unsuppressed | | Suppressed | | New infections |
| --- | --- | --- | --- | --- | --- | --- | --- | --- | --- |
|  | Number | Percentage | Number | Percentage | Number | Percentage | Number | Percentage | Number |
| 2004 | 1880 (1590-2190) | 14.5% (11.2-18.7%) | 4430 (2980-5850) | 34.1% (21-49.8%) | 2750 (1720-3740) | 21.2% (12.2-31.8%) | 3920 (3450-4390) | 30.2% (24.4-37.3%) | 663 (626-695) |
| 2005 | 1860 (1560-2160) | 13.8% (10.6-17.7%) | 4660 (3100-6190) | 34.4% (21-50.6%) | 2770 (1660-3830) | 20.4% (11.2-31.3%) | 4250 (3740-4760) | 31.4% (25.3-38.9%) | 668 (624-704) |
| 2006 | 1830 (1510-2120) | 13% (9.8-16.7%) | 4870 (3200-6520) | 34.7% (20.9-51.5%) | 2190 (950-3380) | 15.6% (6.2-26.7%) | 5150 (4560-5740) | 36.7% (29.7-45.4%) | 670 (620-714) |
| 2007 | 1840 (1510-2130) | 12.6% (9.5-16.2%) | 5460 (3700-7190) | 37.4% (23.2-54.8%) | 1870 (600-3110) | 12.8% (3.8-23.7%) | 5420 (4810-6040) | 37.2% (30.1-46%) | 673 (618-723) |
| 2008 | 1780 (1470-2090) | 11.9% (9-15.5%) | 4210 (2210-6200) | 28.2% (13.5-46.2%) | 2020 (400-3610) | 13.6% (2.5-26.9%) | 6920 (6130-7720) | 46.3% (37.4-57.5%) | 681 (623-734) |
| 2009 | 1730 (1440-2050) | 11.2% (8.5-14.8%) | 4470 (2340-6590) | 28.9% (13.7-47.5%) | 1680 (0-3380) | 10.9% (0-24.4%) | 7560 (6700-8430) | 48.9% (39.4-60.8%) | 696 (643-759) |
| 2010 | 1690 (1420-2010) | 10.6% (8-14%) | 4370 (2070-6660) | 27.3% (11.7-46.3%) | 1620 (0-3500) | 10.1% (0-24.3%) | 8340 (7390-9300) | 52.1% (41.8-64.7%) | 718 (642-794) |
| 2011 | 1650 (1390-1980) | 9.9% (7.5-13.3%) | 4220 (1730-6700) | 25.4% (9.4-45%) | 1640 (0-3720) | 9.9% (0-25%) | 9090 (8050-10160) | 54.8% (43.8-68.2%) | 741 (656-833) |
| 2012 | 1610 (1340-1970) | 9.3% (7-12.8%) | 3460 (720-6200) | 20% (3.7-40.1%) | 1550 (0-3960) | 9% (0-25.6%) | 10640 (9430-11890) | 61.6% (49.2-77%) | 761 (663-859) |
| 2013 | 1560 (1280-1930) | 8.8% (6.4-12.1%) | 3840 (950-6720) | 21.5% (4.8-42.3%) | 1290 (0-3810) | 7.2% (0-24%) | 11130 (9860-12440) | 62.5% (49.7-78.2%) | 773 (675-881) |
| 2014 | 1510 (1210-1890) | 8.2% (5.8-11.5%) | 2800 (0-5980) | 15.1% (0-36.3%) | 1190 (0-4130) | 6.4% (0-25.1%) | 12990 (11510-14520) | 70.2% (55.8-88.2%) | 774 (651-905) |
| 2015 | 1440 (1070-1860) | 7.5% (5-11%) | 2380 (0-5790) | 12.5% (0-34.3%) | 1200 (0-4410) | 6.3% (0-26.1%) | 14050 (12440-15740) | 73.7% (58.3-93.2%) | 759 (567-940) |

These numbers are the input data for the model with the model fitting to the annual number of new infections. All estimates and ranges rounded to the nearest whole number. Percentage for each cascade step is the percentage of all people living with HIV.

**Supplementary Figure S1.** **Number of GBM living with HIV in each step of the HIV cascade over 2004-2015**.


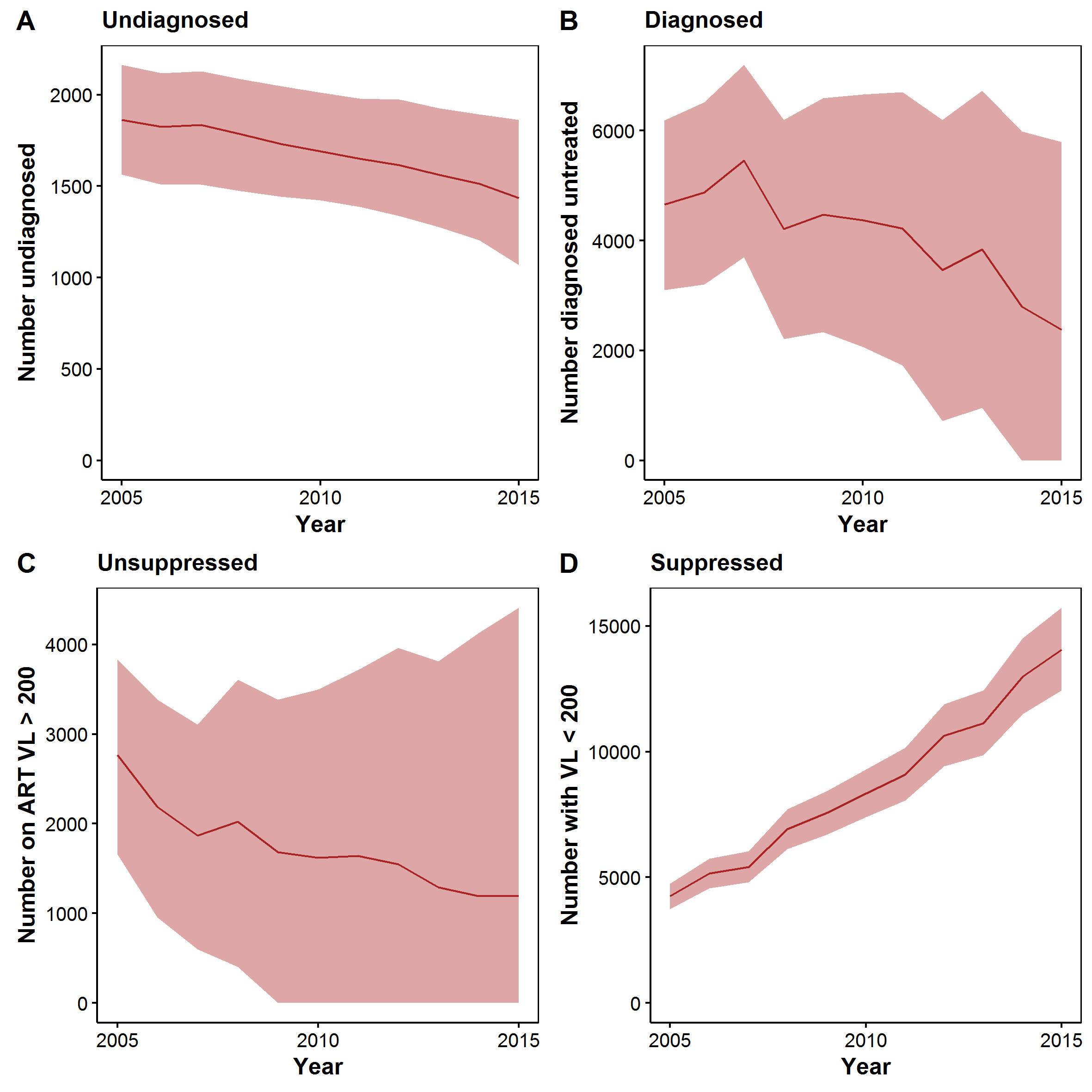


- 1. **Annual new infections attributable to each step of the Australian GBM HIV cascade**

**Supplementary Table S3. New infections attributable to each step of the Australian GBM HIV cascade.**

| **Year** | **Undiagnosed** | | **Diagnosed** | | **Unsuppressed** | | **Suppressed** | | **Total** |
| --- | --- | --- | --- | --- | --- | --- | --- | --- | --- |
|  | Number | Percentage | Number | Percentage | Number | Percentage | Number | Percentage | Number |
| 2004 | 214 (16-523) | 33.4% (2.4-81.2%) | 137 (14-362) | 21.6% (2.1-57.4%) | 280 (29-553) | 43.5% (4.6-84.5%) | 10 (0-36) | 1.5% (0-5.7%) | 640 (555-724) |
| 2005 | 242 (58-518) | 35.4% (8.3-77.3%) | 149 (29-360) | 21.8% (4.1-53.1%) | 285 (50-544) | 41.2% (7.5-76.5%) | 11 (1-37) | 1.6% (0.1-5.4%) | 688 (615-761) |
| 2006 | 267 (88-516) | 39.8% (13.3-75.6%) | 162 (39-364) | 24% (5.8-52.6%) | 228 (49-420) | 34.3% (7.3-64.1%) | 13 (1-43) | 2% (0.2-6.3%) | 678 (612-738) |
| 2007 | 299 (114-529) | 42.8% (16.8-74.3%) | 187 (49-394) | 26.5% (7.3-53.1%) | 198 (48-358) | 28.7% (6.6-54.3%) | 14 (2-43) | 2.1% (0.2-6.1%) | 710 (628-785) |
| 2008 | 320 (129-531) | 45.4% (18.3-74.9%) | 149 (38-299) | 21.2% (5.5-42.8%) | 216 (54-393) | 30.7% (7.9-55.4%) | 19 (2-53) | 2.7% (0.3-7.5%) | 707 (644-768) |
| 2009 | 339 (138-546) | 48% (19.8-76.1%) | 163 (41-314) | 23.1% (6-44.1%) | 182 (46-332) | 25.9% (6.5-48.2%) | 21 (3-57) | 3% (0.4-8.2%) | 709 (638-777) |
| 2010 | 359 (146-560) | 49.5% (20.2-77.3%) | 165 (41-313) | 22.7% (5.8-43.1%) | 177 (43-330) | 24.5% (6-45.8%) | 24 (3-65) | 3.3% (0.4-9%) | 725 (646-805) |
| 2011 | 377 (153-586) | 50.4% (20.3-79%) | 164 (39-312) | 21.8% (5.2-41.9%) | 182 (40-346) | 24.2% (5.5-45.8%) | 26 (3-75) | 3.5% (0.4-10.3%) | 748 (656-843) |
| 2012 | 396 (155-614) | 53.4% (22-82.3%) | 138 (29-269) | 18.8% (3.9-37.9%) | 173 (34-341) | 23.4% (4.7-46.1%) | 32 (3-96) | 4.3% (0.5-13%) | 732 (628-848) |
| 2013 | 408 (150-636) | 54.6% (21.2-83.9%) | 157 (29-315) | 21.1% (3.8-42.8%) | 146 (24-297) | 19.7% (3.2-41.5%) | 34 (3-109) | 4.5% (0.4-14.7%) | 738 (619-868) |
| 2014 | 421 (142-668) | 58.2% (22.2-87.6%) | 118 (18-246) | 16.8% (2.4-36.7%) | 136 (16-289) | 19.3% (2.2-42.7%) | 40 (3-136) | 5.6% (0.4-18.6%) | 700 (566-855) |
| 2015 | 423 (132-680) | 59% (20.8-89.8%) | 103 (8-221) | 15% (1.2-34.4%) | 138 (6-307) | 19.8% (0.9-45.3%) | 44 (1-159) | 6.2% (0.2-21.4%) | 691 (543-861) |

Estimated number (mean and 95% CrI rounded to the nearest whole number) and percentage (mean and 95% credible interval) of new infections attributable to each step of the Australian GBM HIV cascade over 2004-2015.

- 2. **Transmission Coefficient Priors and Posteriors for Main Results**

**Supplementary Figure S2. Priors and resulting posteriors for main analysis.**


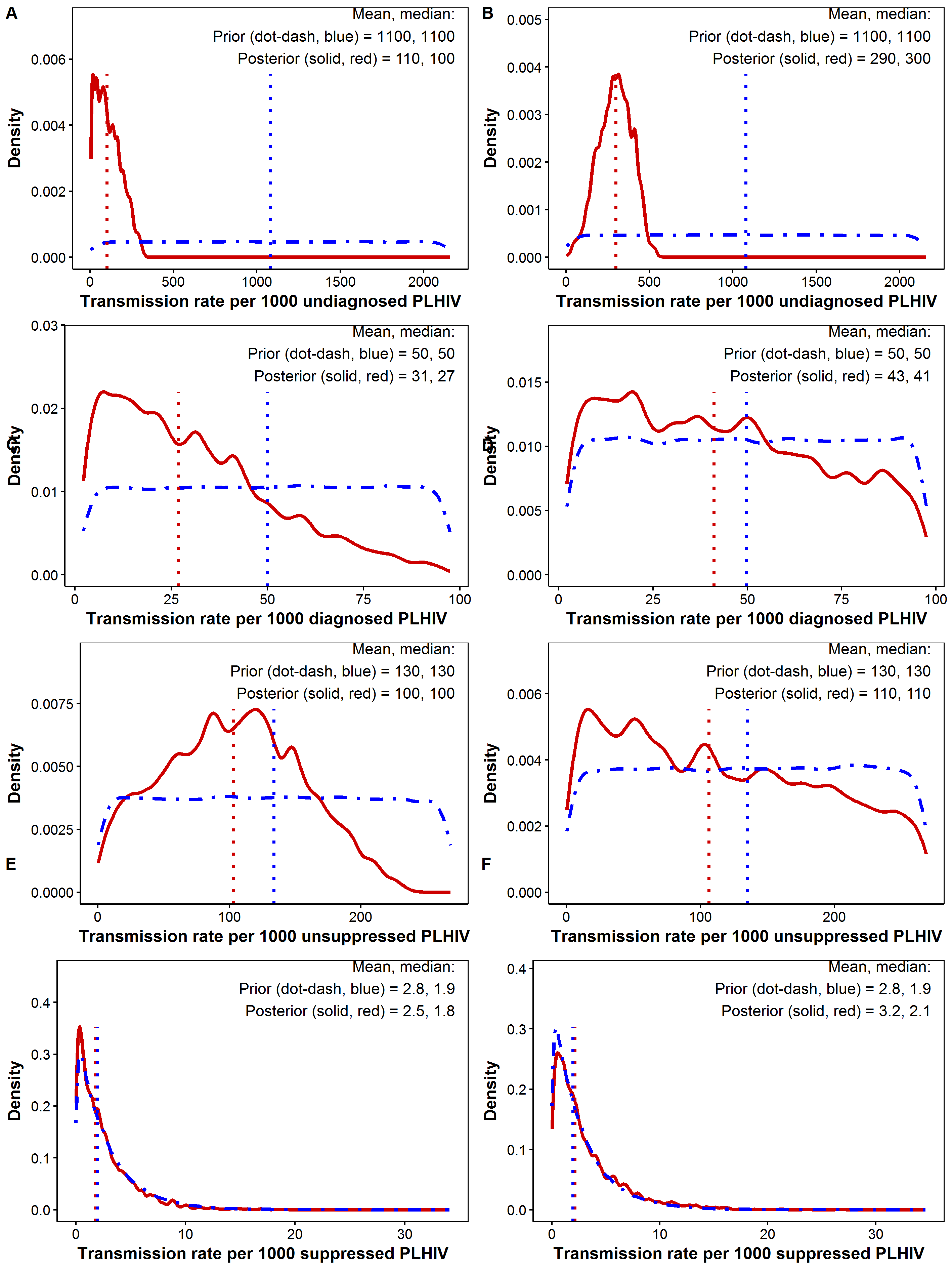


Priors (blue) and resulting posteriors (red) for each parameter in the model (Eq. 1 of the main text). The left column shows the parameter distributions for the start of the analysis (in 2004) and the right column shows the parameter distributions for the end of the analysis (in 2015). The dashed lines show the median of the distributions. Note the rounding at the ends of the uniform priors is an artefact of plotting the density of the prior sample.

**Supplementary Figure S3. Posterior distributions for the transmission rate for each step of the GBM HIV cascade at the start and end of the analysis period, 2004-2015.**


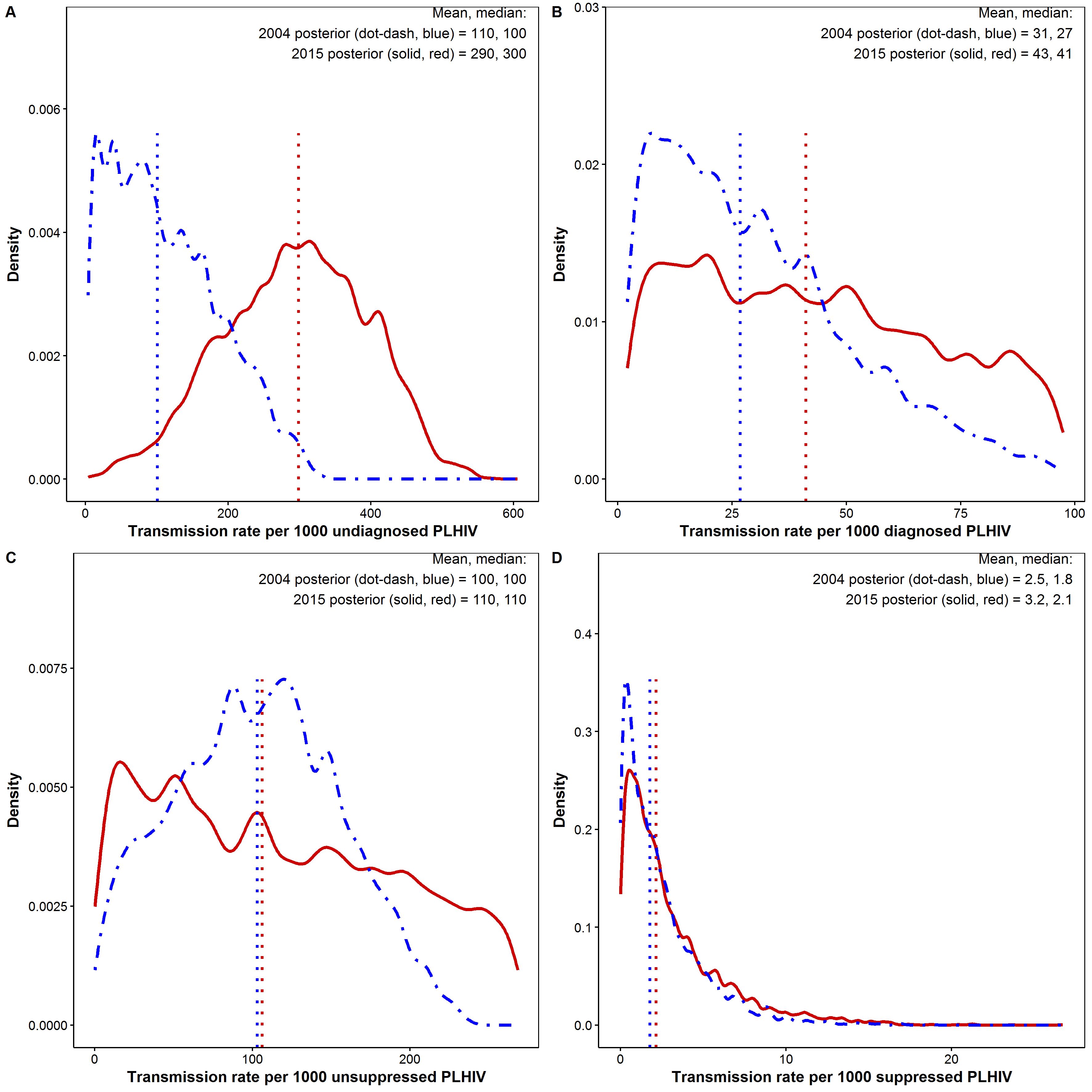


The dashed lines show the median of the distributions.

**Supplementary Figure S4.** **The posterior distributions for the transmission coefficients for each step of the GBM HIV cascade in 2004 A) and 2015 B) on a log10 scale.**

**A**


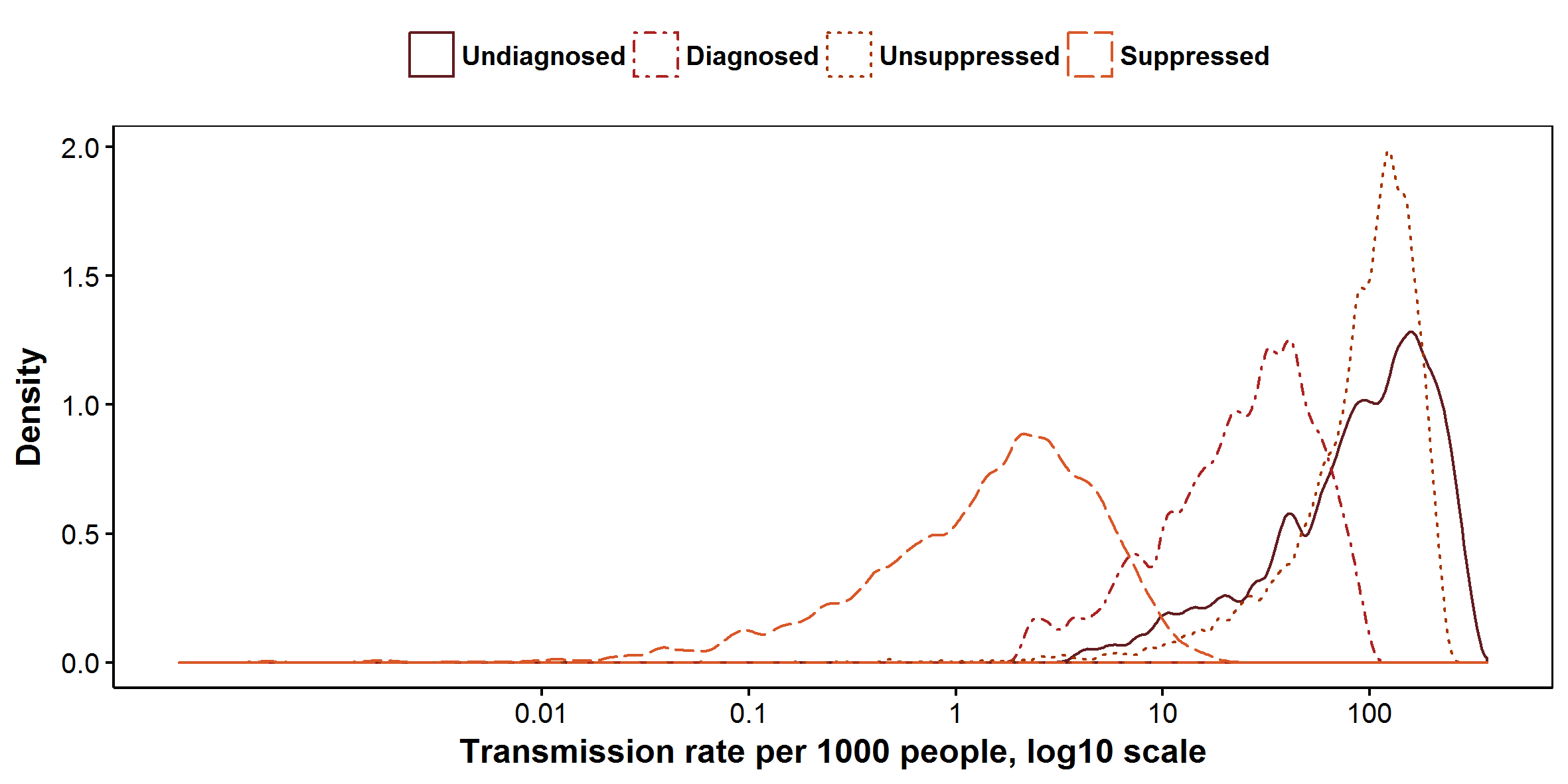


**B**


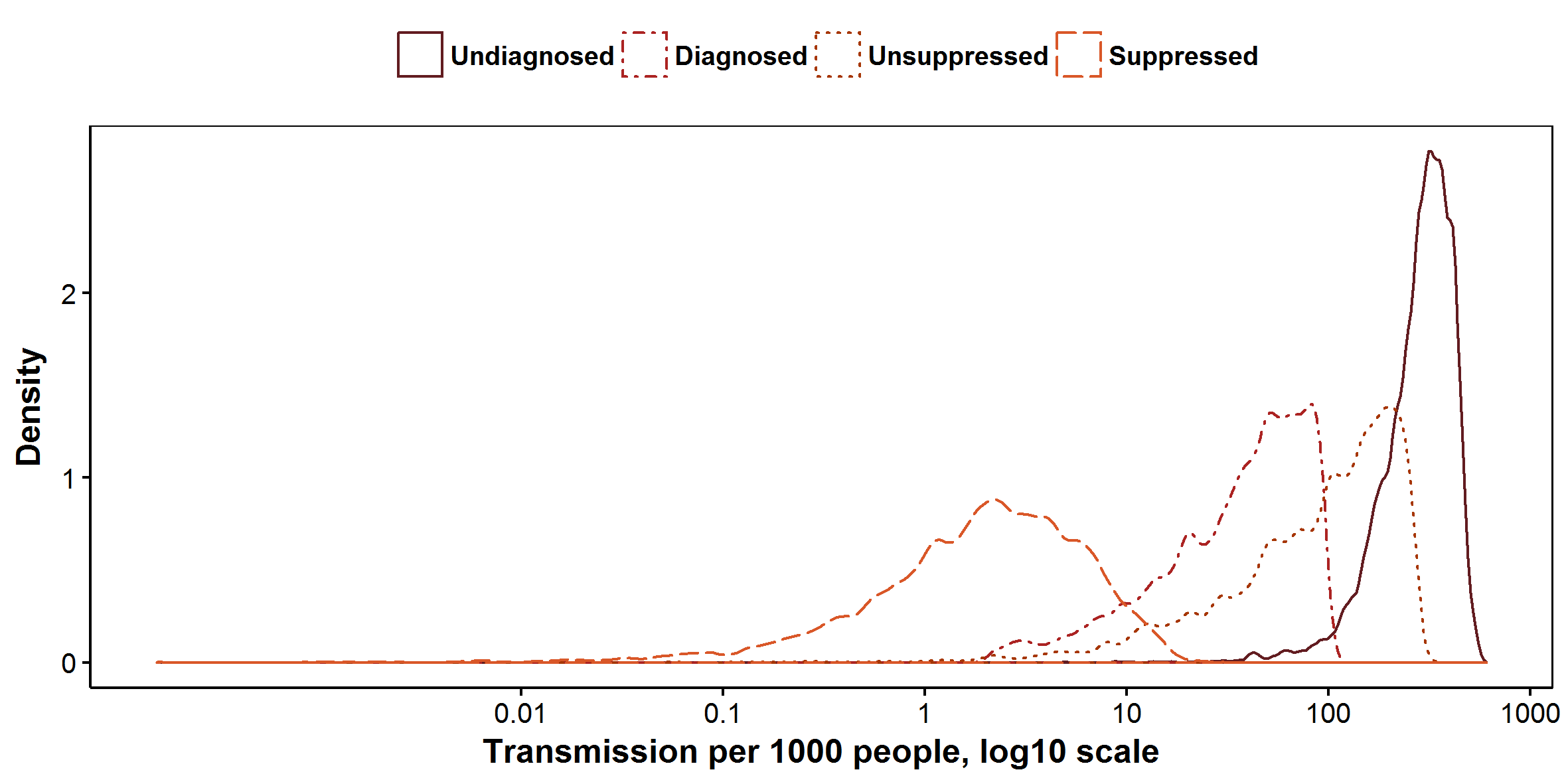


**Supplementary Figure S5: Bilinear correlation corner plots for transmission coefficient posteriors in (A) 2004 (start) and (B) 2015 (end).**

**A**


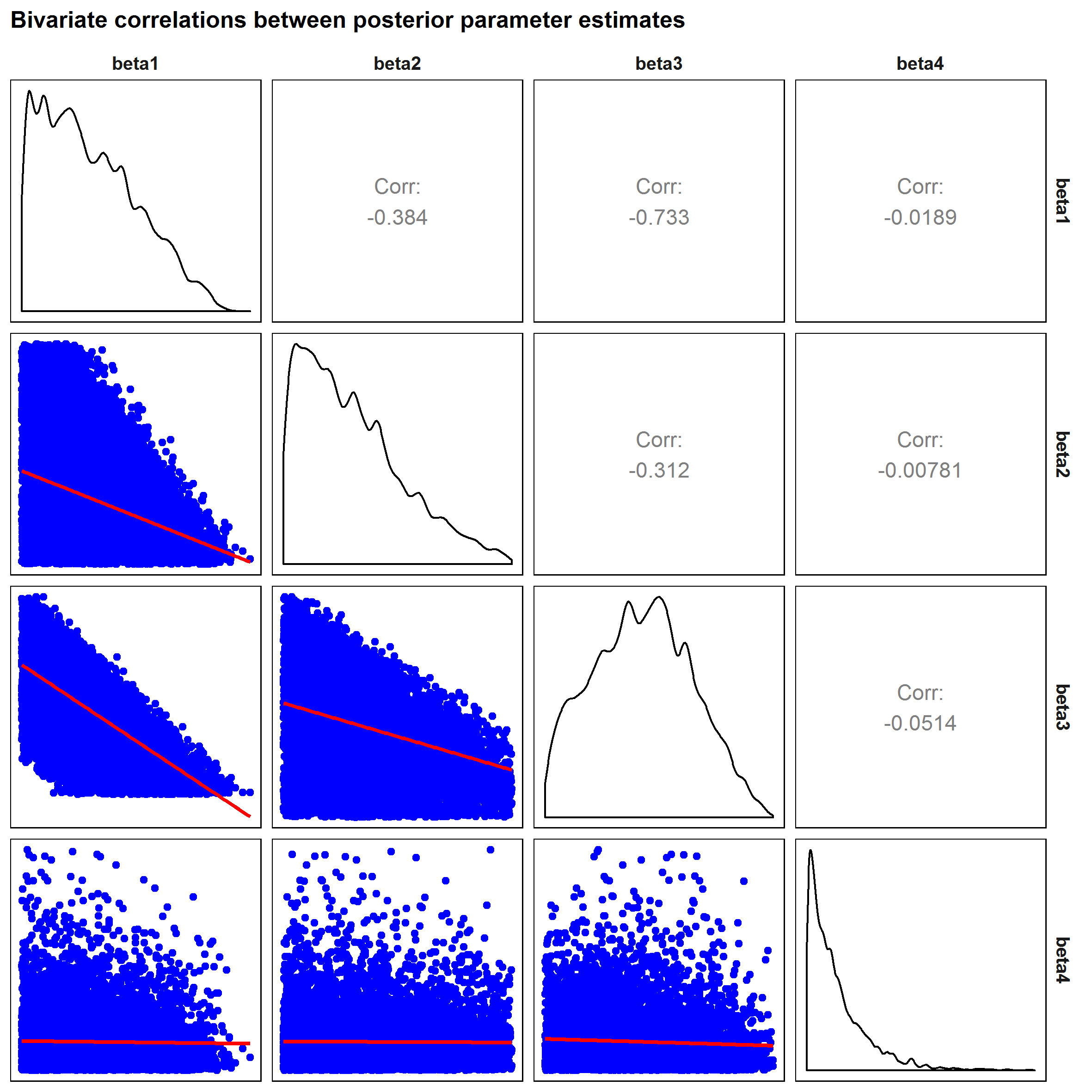


**B**

**
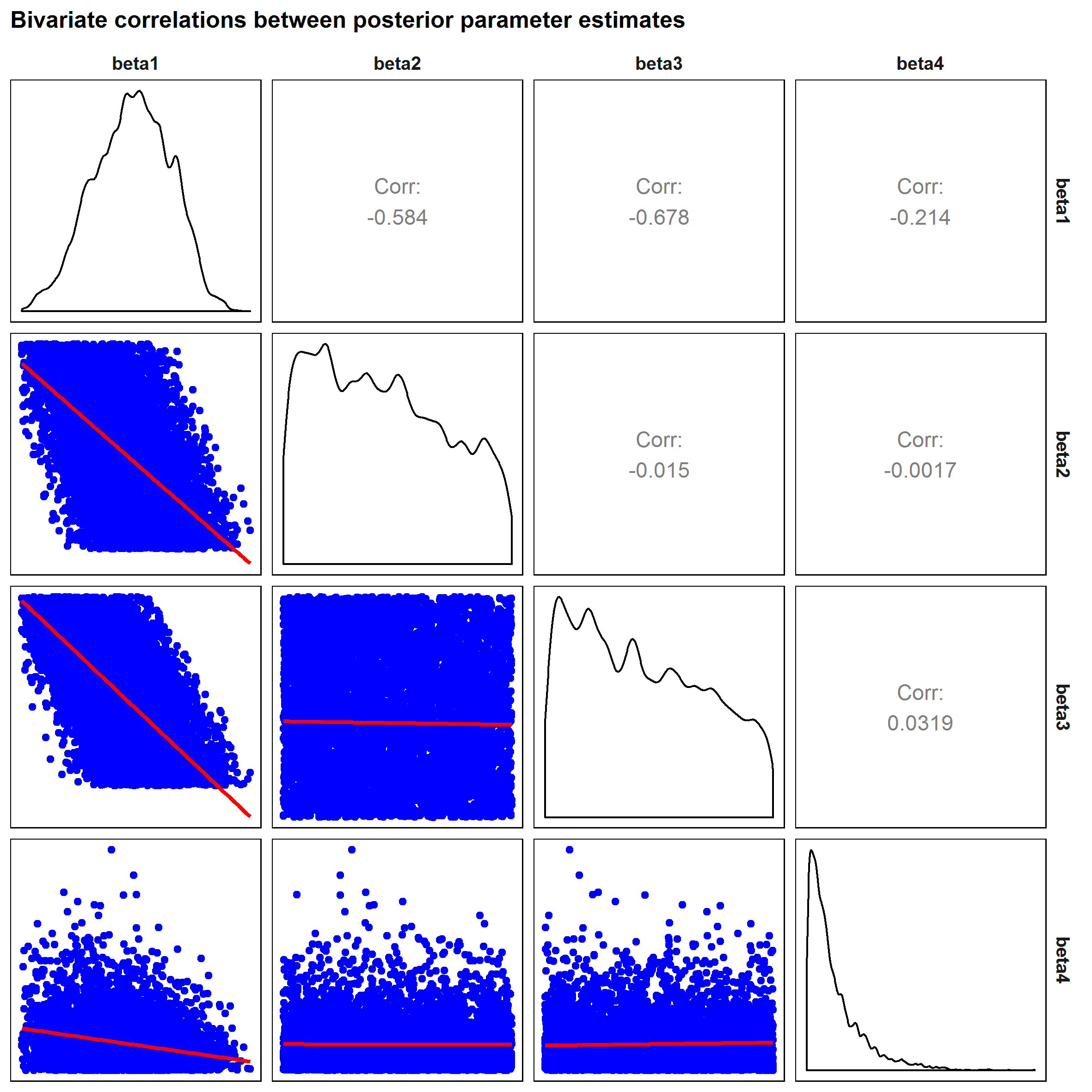
**

- 1. **transmission Coefficient estimates for main results**

**Supplementary Table S4. Posterior rates of transmission in 2004 and 2015 per 1000 people in each step of the HIV cascade.**

|  | 2004 | | 2015 | |
| --- | --- | --- | --- | --- |
| Step | Value | Relative to diagnosed | Value | Relative to diagnosed |
| Undiagnosed | 110 (8.5-280) | 8.9 (0.19-51) | 290 (92-470) | 17 (1.2-100) |
| Diagnosed | 31 (3.1-82) | 1 (1-1) | 43 (3.6-93) | 1 (1-1) |
| Unsuppressed | 100 (10-200) | 7.4 (0.29-41) | 120 (5-260) | 5.8 (0.12-36) |
| Suppressed | 2.5 (0.067-9.1) | 0.16 (0.002-0.97) | 3.2 (0.093-11) | 0.15 (0.0022-1) |

Mean and 95% credible interval of the posterior rate of transmission in 2004 and 2015 per 1000 people in each step of the HIV cascade. Results rounded to two significant figures.

1. **Sensitivity Analysis results**

In this section all the results from the sensitivity analysis scenarios described in Section 1.3 are provided. Table S5 provides a comparison of the transmission coefficient and percentage contribution to new infections for each step of the Australian HIV cascade. The following sections provide the full results for each scenario.

**Supplementary Table S5: Transmission coefficients and percentage of new HIV infections attributable to each step of the HIV cascade for each analysis scenario in 2004 and final year of analysis (2014, 2015).**

|  |  | **Undiagnosed** | | **Diagnosed** | | **Unsuppressed** | | **Suppressed** | |
| --- | --- | --- | --- | --- | --- | --- | --- | --- | --- |
| Scenario | Year | Transmission coefficient (per 1000 people; mean,95% CrI) | Percentage of new infections  mean,95% CrI | Transmission coefficient (per 1000 people; mean,95% CrI) | Percentage of new infections  mean,95% CrI | Transmission coefficient (per 1000 people; mean,95% CrI) | Percentage of new infections  mean,95% CrI | Transmission coefficient (per 1000 people; mean,95% CrI) | Percentage of new infections  mean,95% CrI |
| 2004-2015 cascade with Partner study suppressed prior (main text results) | 2004 | 110 (8.7-280) | 33.2% (2.4-80.8%) | 136 (13-360) | 21.4% (2-57.3%) | 100 (11-200) | 43.9% (4.6-84.6%) | 2.5 (0.059-9) | 1.5% (0-5.6%) |
|  | 2015 | 290 (92-470) | 59.1% (20.9-89%) | 103 (9-222) | 15% (1.2-34.3%) | 110 (5.2-260) | 19.7% (0.9-45.8%) | 3.2 (0.086-12) | 6.2% (0.2-22.5%) |
| 2004-2015 cascade with Cohen 2011 suppressed prior | 2004 | 110 (7.6-280) | 33.3% (2.2-81.3%) | 29 (3.2-7.8) | 20.5% (2.1-55%) | 110 (11-200) | 44.8% (4.8-84.9%) | 2.2 (0.048-12) | 1.4% (0-7.4%) |
|  | 2015 | 290 (64-480) | 57.5% (13.9-91.1%) | 46 (4.3-94) | 15.8% (1.4-33.8%) | 120 (4.7-260) | 19.7% (0.8-45.2%) | 3.6 (0.071-20) | 7% (0.1-36.8%) |
| 2004-2015 cascade with zero suppressed transmission | 2004 | 110 (8.4-270) | 31.8% (2.4-80.9%) | 31 (3.1-8.4) | 21.3% (2.2-57.5%) | 110 (11-200) | 46.9% (5-86.3% | 0 (0-0) | 0% (0-0%) |
|  | 2015 | 320 (120-500) | 65.3% (28-93.1%) | 44 (3.8-94) | 15.2% (1.2-34.8%) | 110 (4.8-260) | 19.5% (0.8-46.4%) | 0 (0-0) | 0% (0-0%) |
| 2004-2015 cascade with no range in cascade estimates and Partner study suppressed prior | 2004 | 160 (45-290) | 47.7% (13.4-83.3%) | 30 (6.3-58) | 20.9% (4.3-40.9%) | 69 (5.8-120) | 29.8% (2.5-51.3%) | 2.6 (0.076-9.6) | 1.6% (0-5.9%) |
|  | 2015 | 370 (240-490) | 73.6% (51.3-91.7%) | 31 (6.2-58) | 10.4% (2-20.8%) | 57 (2.7-120) | 9.7% (0.4-20.4% | 3.2 (0.093-11) | 6.2% (0.2-21.4%) |
| 2004-2014 cascade with Cohen 2011 suppressed prior | 2004 | 120 (10-270) | 35.7% (3.1-82%) | 31 (4.6-71) | 22.3% (3.2-53.2%) | 94 (6.1-190) | 40.6% (2.7-79.6%) | 2.2 (0.06-12) | 1.5% (0-7.7%) |
|  | 2014 | 270 (74-450) | 61.3% (19.3-91.7%) | 38 (5.2-75) | 15.5% (2-32.8%) | 93 (4.3-200) | 18% (0.7-40.9%) | 2.9 (0.07-17) | 5.1% (0.1-30.8%) |

Transmission coefficients rounded to two significant figures.

- 1. **Australian GBM HIV cascade during 2004-2015 with Cohen 2011 suppressed transmision coefficint prior**

This section shows the results obtained by our methodology when using the Australian GBM HIV cascade over 2004-2015 (obtained using the 2015 HIV cascade methodology [1]) with the transmission coefficient for the suppressed population having a prior based on the results from the Cohen et al study in 2011. This prior is given by a lognormal distribution with mean 0.04 (95% CI: 0.01-0.27) [12] and describes the relative reduction in the transmission coefficient compared to the diagnosed not on ART population, as shown in Eq. 2.

**Supplementary Figure S6. Estimated number and percentage of new infections attributed to each step of the Australian GBM HIV cascade.**


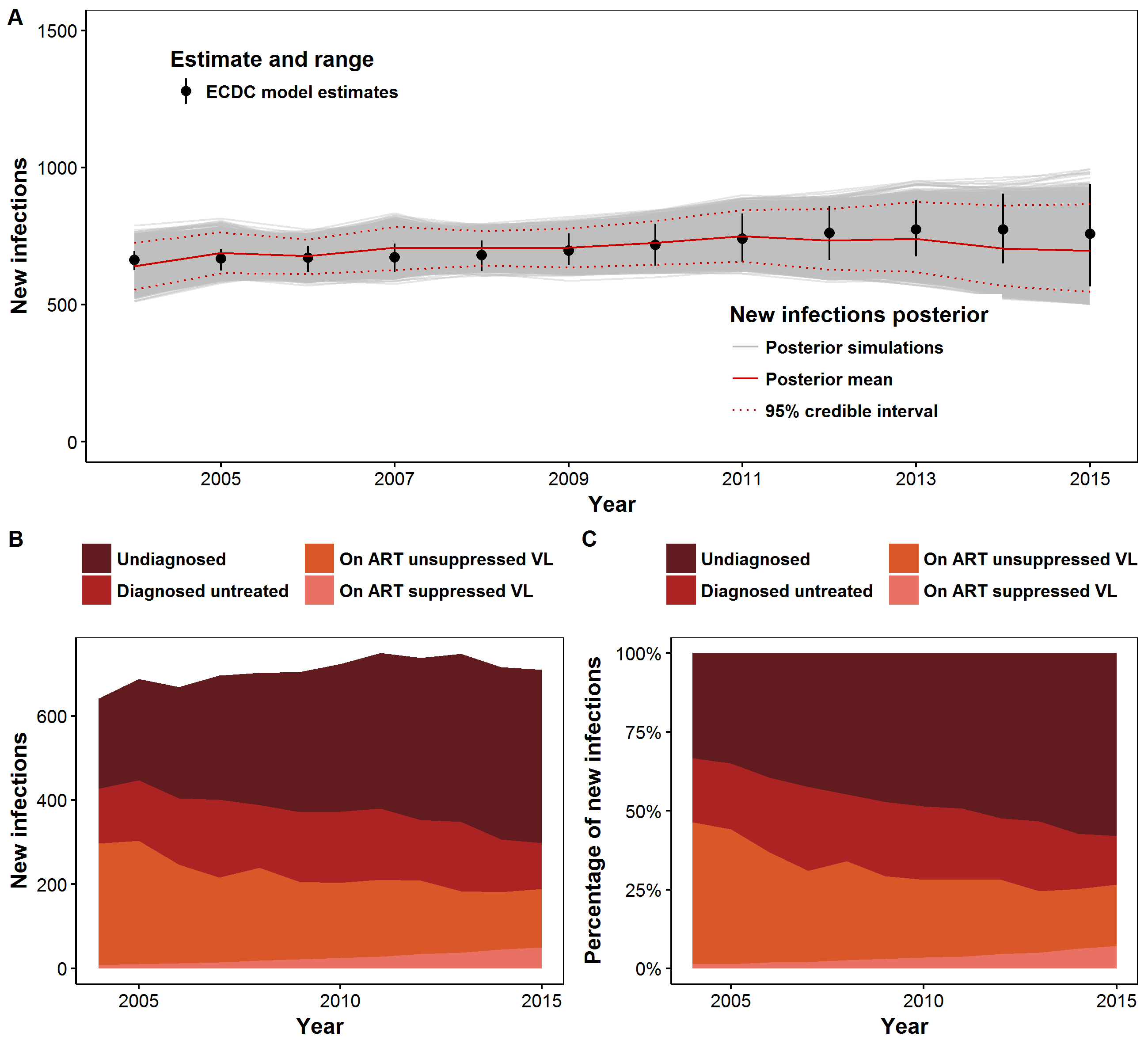


**A)** Estimated new infections for the posterior simulations for the Australian GBM HIV cascade during 2004-2015 with Cohen 2011 suppressed transmission coefficient prior and using the 2015 HIV cascade methodology. Each thin grey line is one simulation in the posterior, the thick red line is the posterior mean value at each time point and the dashed red lines are the lower and upper bounds of the 95% credible interval. The black dots and lines show the estimated number new infections and range produced by the ECDC HIV Modelling Tool. Estimated number **B)** and proportion **C)** of overall new infections attributed to each step of the GBM HIV cascade.

**Supplementary Figure S7. The posterior distribution in percentage of new infections attributed to each step of the Australian GBM HIV cascade during 2004-2015 with Cohen 2011 suppressed transmission coefficient prior and using the 2015 HIV cascade methodology.**


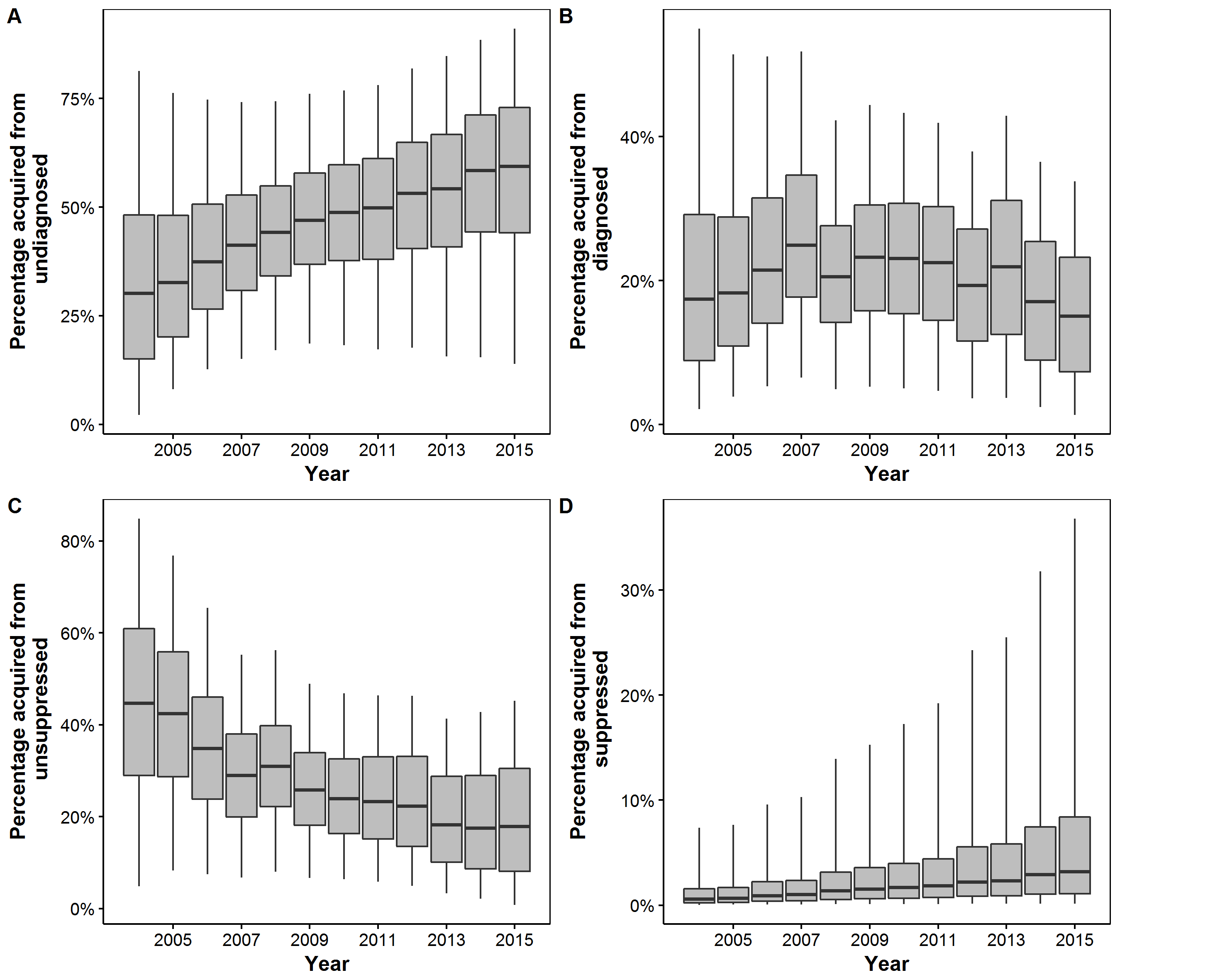


**Supplementary Table S6. New infections attributable to each step of the Australian GBM HIV cascade.**

| **Year** | **Undiagnosed** | | **Diagnosed** | | **Unsuppressed** | | **Suppressed** | | **Total** |
| --- | --- | --- | --- | --- | --- | --- | --- | --- | --- |
|  | Number | Percentage | Number | Percentage | Number | Percentage | Number | Percentage |  |
| 2004 | 213 (14-524) | 33.3% (2.2-81.3%) | 130 (14-344) | 20.5% (2.1-55%) | 289 (31-557) | 44.8% (4.8-84.9%) | 9 (0-48) | 1.4% (0-7.4%) | 640 (555-726) |
| 2005 | 241 (57-515) | 35.2% (8.1-76.2%) | 144 (28-350) | 21% (3.9-51.5%) | 293 (55-548) | 42.4% (8.3-76.8%) | 10 (0-52) | 1.5% (0-7.7%) | 688 (615-762) |
| 2006 | 264 (85-511) | 39.4% (12.6-74.8%) | 158 (36-353) | 23.5% (5.3-51.1%) | 234 (52-422) | 35.2% (7.5-65.4%) | 13 (0-64) | 1.9% (0.1-9.6%) | 677 (612-737) |
| 2007 | 295 (106-529) | 42.3% (15.1-74.1%) | 185 (43-384) | 26.4% (6.5-51.9%) | 202 (50-363) | 29.3% (6.7-55.3%) | 14 (0-70) | 2% (0.1-10.3%) | 708 (626-783) |
| 2008 | 314 (121-532) | 44.7% (17-74.4%) | 149 (34-293) | 21.3% (4.9-42.2%) | 220 (56-400) | 31.3% (8-56.2%) | 19 (1-96) | 2.7% (0.1-13.9%) | 706 (642-767) |
| 2009 | 332 (126-542) | 47.1% (18.6-76.1%) | 166 (37-314) | 23.5% (5.3-44.4%) | 185 (47-343) | 26.3% (6.6-48.9%) | 22 (1-107) | 3.1% (0.1-15.2%) | 709 (636-777) |
| 2010 | 351 (131-563) | 48.5% (18.2-76.9%) | 168 (36-314) | 23.3% (5-43.3%) | 179 (46-337) | 24.8% (6.4-46.9%) | 25 (1-122) | 3.4% (0.1-17.2%) | 726 (645-805) |
| 2011 | 369 (127-591) | 49.3% (17.3-78.1%) | 169 (34-315) | 22.6% (4.7-41.9%) | 183 (44-352) | 24.4% (5.8-46.4%) | 28 (1-145) | 3.8% (0.1-19.2%) | 749 (657-845) |
| 2012 | 386 (127-623) | 52.2% (17.6-81.9%) | 144 (28-270) | 19.6% (3.7-37.9%) | 174 (37-346) | 23.6% (5-46.3%) | 34 (1-180) | 4.6% (0.1-24.3%) | 734 (627-849) |
| 2013 | 398 (114-647) | 53.2% (15.7-84.8%) | 166 (27-316) | 22.1% (3.7-42.9%) | 146 (26-300) | 19.8% (3.3-41.3%) | 37 (1-198) | 4.9% (0.1-25.5%) | 741 (620-874) |
| 2014 | 410 (105-676) | 56.8% (15.5-88.5%) | 125 (18-246) | 17.7% (2.4-36.5%) | 136 (16-290) | 19.3% (2.2-42.8%) | 45 (1-243) | 6.2% (0.1-31.8%) | 704 (568-861) |
| 2015 | 411 (92-688) | 57.5% (13.9-91.1%) | 110 (10-223) | 15.8% (1.4-33.8%) | 138 (6-308) | 19.7% (0.8-45.2%) | 50 (1-282) | 7% (0.1-36.8%) | 696 (547-867) |

Estimated number (mean and 95% CrI rounded to the nearest whole number) and percentage (mean and 95% credible interval) of new infections attributable to each step of the Australian GBM HIV cascade over 2004-2015 with Cohen 2011 suppressed transmission coefficient prior and using the 2015 HIV cascade methodology.

**Supplementary Figure S8.** **Change in the rate of transmission for people living with HIV in each step of the Australian GBM HIV cascade during 2004-2015 with Cohen 2011 suppressed transmission coefficient prior and using the 2015 HIV cascade methodology.**


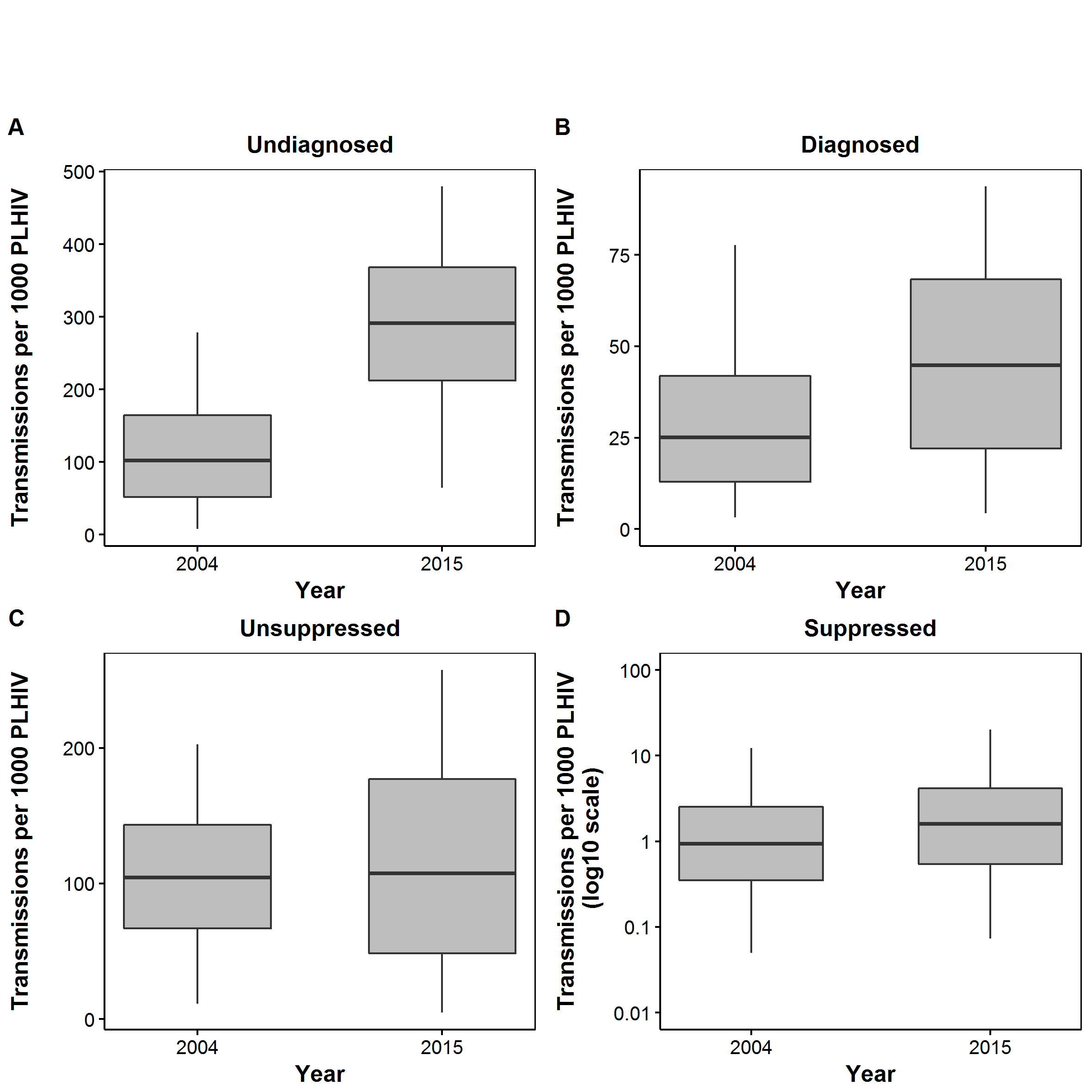


**Supplementary Table S7. Posterior rate of transmission in 2004 and 2015 per 1000 people in each step of the HIV cascade.**

|  | 2004 | | 2015 | |
| --- | --- | --- | --- | --- |
| Step | Value | Relative to diagnosed | Value | Relative to diagnosed |
| Undiagnosed | 110 (7.6-280) | 9.5 (0.18-57) | 290 (64-480) | 15 (0.8-90) |
| Diagnosed | 29 (3.2-78) | 1 (1-1) | 46 (4.3-94) | 1 (1-1) |
| Unsuppressed | 110 (11-200) | 7.9 (0.3-40) | 120 (4.7-260) | 5.2 (0.1-32) |
| Suppressed | 2.2 (0.048-12) | 0.078 (0.0036-0.38) | 3.6 (0.071-20) | 0.078 (0.0036-0.38) |

Mean and 95% credible interval of the posterior rates of transmission in 2004 and 2015 per 1000 people in each step of the Australian GBM HIV cascade during 2004-2015 with Cohen 2011 suppressed transmission coefficient prior and using the 2015 HIV cascade methodology. Results rounded to two significant figures.

- 1. **Australian GBM HIV cascade During 2004-2015 with zero suppressed transmission**

This section shows the results obtained by our methodology when using the Australian GBM HIV cascade during 2004-2015 (obtained using the 2015 HIV cascade methodology [1]) with the transmission coefficient for the suppressed population equal to zero (i.e. no transmission from the suppressed population).

**Supplementary Figure S9.** **Estimated number and percentage of new infections attributed to each step of the Australian GBM HIV cascade.**


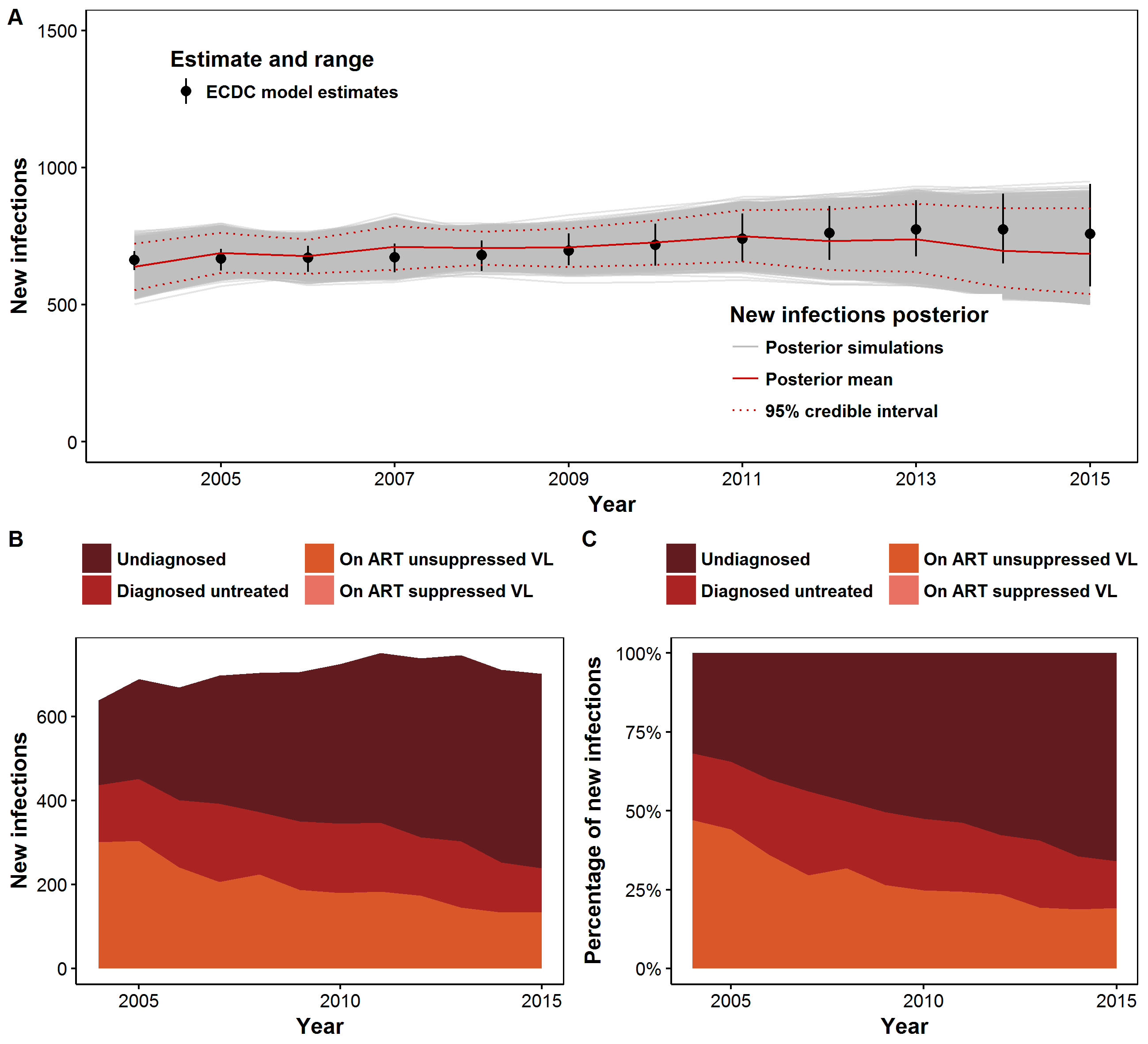


**A)** Estimated new infections for the posterior simulations for the Australian GBM HIV cascade during 2004-2015 with zero suppressed transmission and the 2015 HIV cascade methodology. Each thin grey line is one simulation in the posterior, the thick red line is the posterior mean value at each time point and the dashed red lines are the lower and upper bounds of the 95% credible interval. The black dots and lines show the estimated number new infections and range produced by the ECDC HIV Modelling Tool. Estimated number **B)** and proportion **C)** of overall new infections attributed to each step of the GBM HIV cascade.

**Supplementary Figure S10.** **The posterior distribution in percentage of new infections attributed to each step of the Australian GBM HIV cascade over 2004-2015 with zero suppressed transmission.**


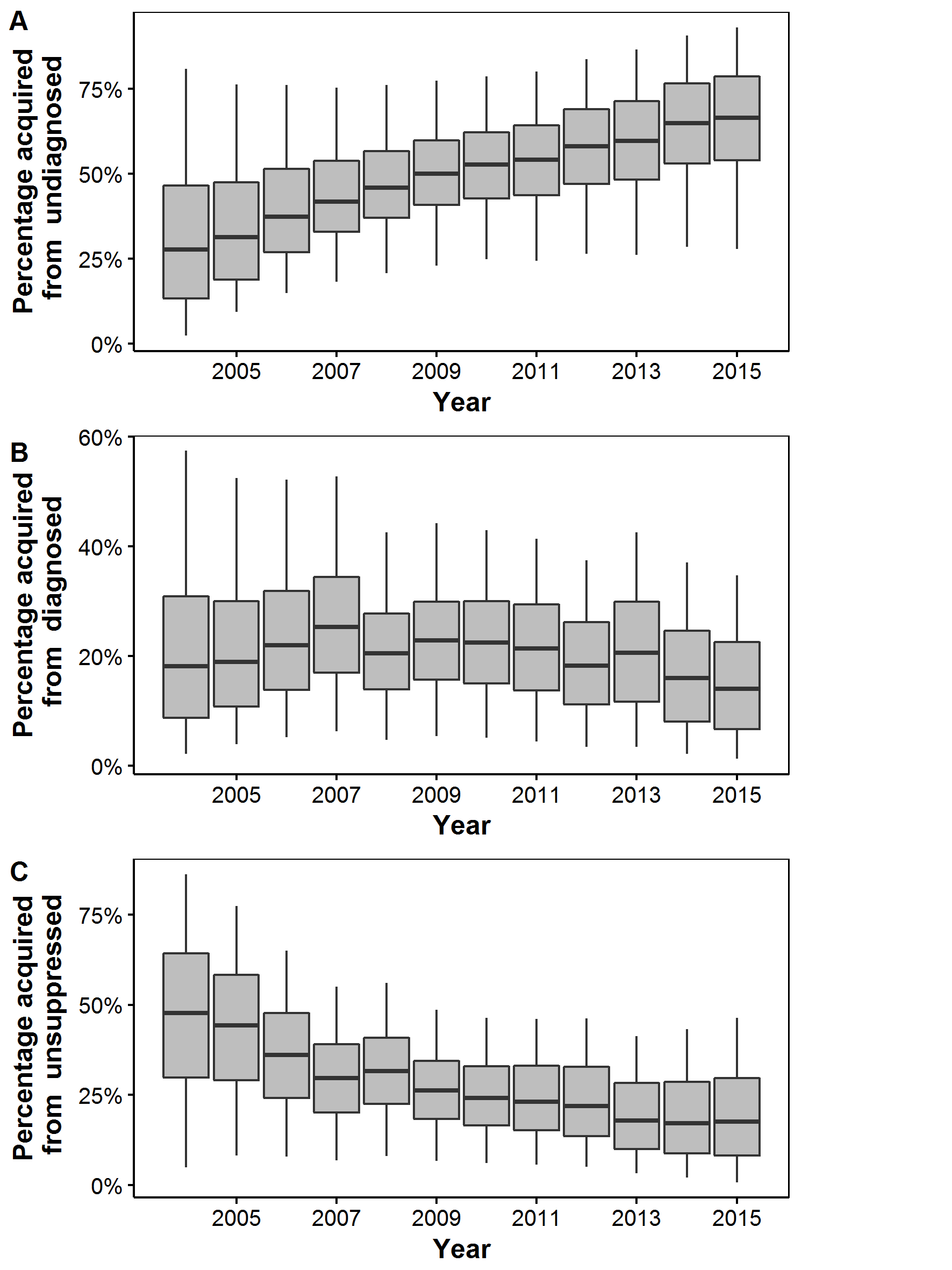


**Supplementary Table S8.** **New infections attributable to each step of the Australian GBM HIV cascade.**

| **Year** | **Undiagnosed** | | **Diagnosed** | | **Unsuppressed** | | **Suppressed** | | **Total** |
| --- | --- | --- | --- | --- | --- | --- | --- | --- | --- |
|  | Number | Percentage | Number | Percentage | Number | Percentage | Number | Percentage |  |
| 2004 | 203 (16-514) | 31.8% (2.4-80.9%) | 135 (14-357) | 21.3% (2.2-57.5%) | 301 (31-562) | 46.9% (5-86.3%) | 0 (0-0) | 0% (0-0%) | 638 (554-723) |
| 2005 | 237 (65-516) | 34.6% (9.3-76.3%) | 148 (27-357) | 21.5% (3.9-52.5%) | 304 (55-546) | 43.8% (8.2-77.4%) | 0 (0-0) | 0% (0-0%) | 689 (616-762) |
| 2006 | 268 (99-517) | 40% (14.8-76.2%) | 161 (35-359) | 23.8% (5.2-52.2%) | 241 (54-424) | 36.2% (7.9-65%) | 0 (0-0) | 0% (0-0%) | 678 (612-736) |
| 2007 | 306 (128-534) | 43.7% (18.2-75.3%) | 186 (41-390) | 26.4% (6.2-52.8%) | 206 (50-362) | 30% (6.9-55.1%) | 0 (0-0) | 0% (0-0%) | 711 (627-787) |
| 2008 | 332 (146-543) | 47% (20.7-76.2%) | 149 (34-294) | 21.2% (4.7-42.6%) | 224 (57-398) | 31.8% (8.1-56%) | 0 (0-0) | 0% (0-0%) | 707 (645-766) |
| 2009 | 356 (162-559) | 50.3% (23-77.5%) | 163 (38-315) | 23.1% (5.4-44.2%) | 187 (48-338) | 26.6% (6.7-48.7%) | 0 (0-0) | 0% (0-0%) | 710 (637-777) |
| 2010 | 381 (177-581) | 52.4% (24.9-78.7%) | 165 (36-314) | 22.7% (5.1-43%) | 180 (45-335) | 24.9% (6.1-46.4%) | 0 (0-0) | 0% (0-0%) | 727 (645-806) |
| 2011 | 404 (183-604) | 53.8% (24.4-80.1%) | 164 (33-312) | 21.9% (4.4-41.4%) | 183 (43-352) | 24.3% (5.7-46.1%) | 0 (0-0) | 0% (0-0%) | 750 (656-845) |
| 2012 | 426 (189-638) | 57.6% (26.4-83.7%) | 139 (26-267) | 19% (3.4-37.4%) | 173 (36-344) | 23.4% (5-46.3%) | 0 (0-0) | 0% (0-0%) | 732 (626-846) |
| 2013 | 443 (187-669) | 59.2% (26.2-86.5%) | 159 (26-314) | 21.3% (3.5-42.6%) | 145 (25-299) | 19.5% (3.3-41.3%) | 0 (0-0) | 0% (0-0%) | 738 (620-868) |
| 2014 | 459 (184-702) | 63.9% (28.5-90.7%) | 119 (17-245) | 17.1% (2.2-37.1%) | 134 (15-289) | 19.1% (2.1-43.2%) | 0 (0-0) | 0% (0-0%) | 697 (562-851) |
| 2015 | 463 (175-719) | 65.3% (28-93.1%) | 104 (9-223) | 15.2% (1.2-34.8%) | 135 (6-306) | 19.5% (0.8-46.4%) | 0 (0-0) | 0% (0-0%) | 686 (538-852) |

Estimated number (mean and 95% CrI rounded to the nearest whole number) and percentage (mean and 95% credible interval) of new infections attributable to each step of the Australian GBM HIV cascade during 2004-2015 with zero suppressed transmission.

**Supplementary Figure S11.** **Change in the rate of transmission for people living with HIV in each step of the Australian GBM HIV cascade over 2004-2015 with zero suppressed transmission.**


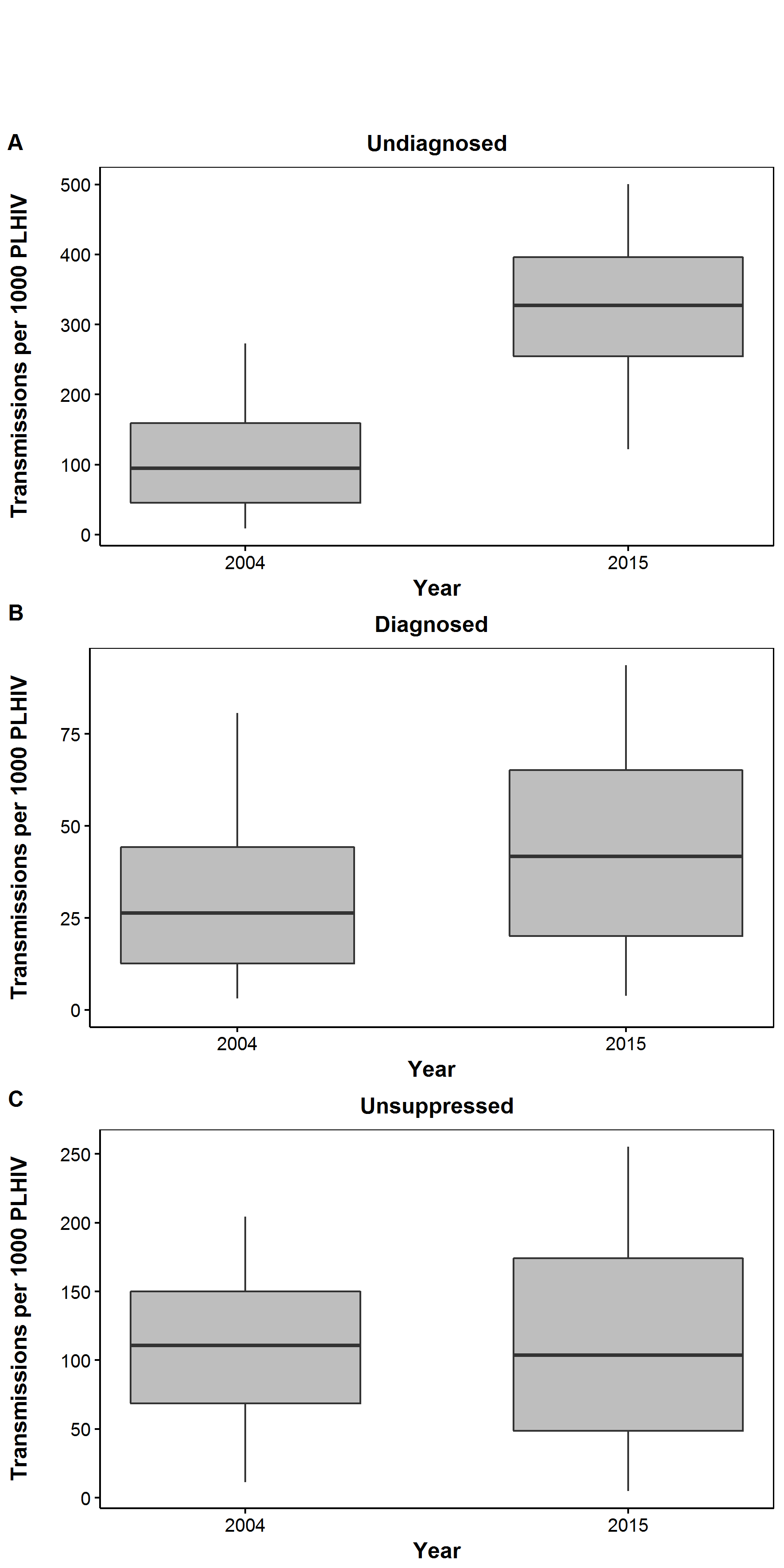


**Supplementary Table S9. Posterior rates of transmission in 2004 and 2015 per 1000 people in each step of the HIV cascade.**

|  | 2004 | | 2015 | |
| --- | --- | --- | --- | --- |
| Step | Value | Relative to diagnosed | Value | Relative to diagnosed |
| Undiagnosed | 110 (8.4-270) | 8.9 (0.18-52) | 320 (120-500) | 18 (1.5-110) |
| Diagnosed | 31 (3.1-81) | 1 (1-1) | 44 (3.8-94) | 1 (1-1) |
| Unsuppressed | 110 (11-200) | 8.4 (0.3-45) | 110 (4.8-260) | 5.6 (0.11-33) |
| Suppressed | 0 (0-0) | 0 (0-0) | 0 (0-0) | 0 (0-0) |

Mean and 95% credible interval of the posterior rates of transmission in 2004 and 2015 per 1000 people in each step of the Australian GBM HIV cascade during 2004-2015 with zero suppressed transmission. Results rounded to two significant figures.

- 1. **Australian GBM HIV cascade During 2004-2015 with Partner study Suppressed Transmission coefficient and no range in cascade estimates**

This section shows the results obtained by our methodology when using the Australian GBM HIV cascade during 2004-2015 (obtained using the 2015 HIV cascade methodology [1]) with the transmission coefficient for the suppressed population having a prior based on the results from the Partner study (as for the results in the main text)[11].

**Supplementary Figure S12. Estimated number and percentage of new infections attributed to each step of the Australian GBM HIV cascade.**


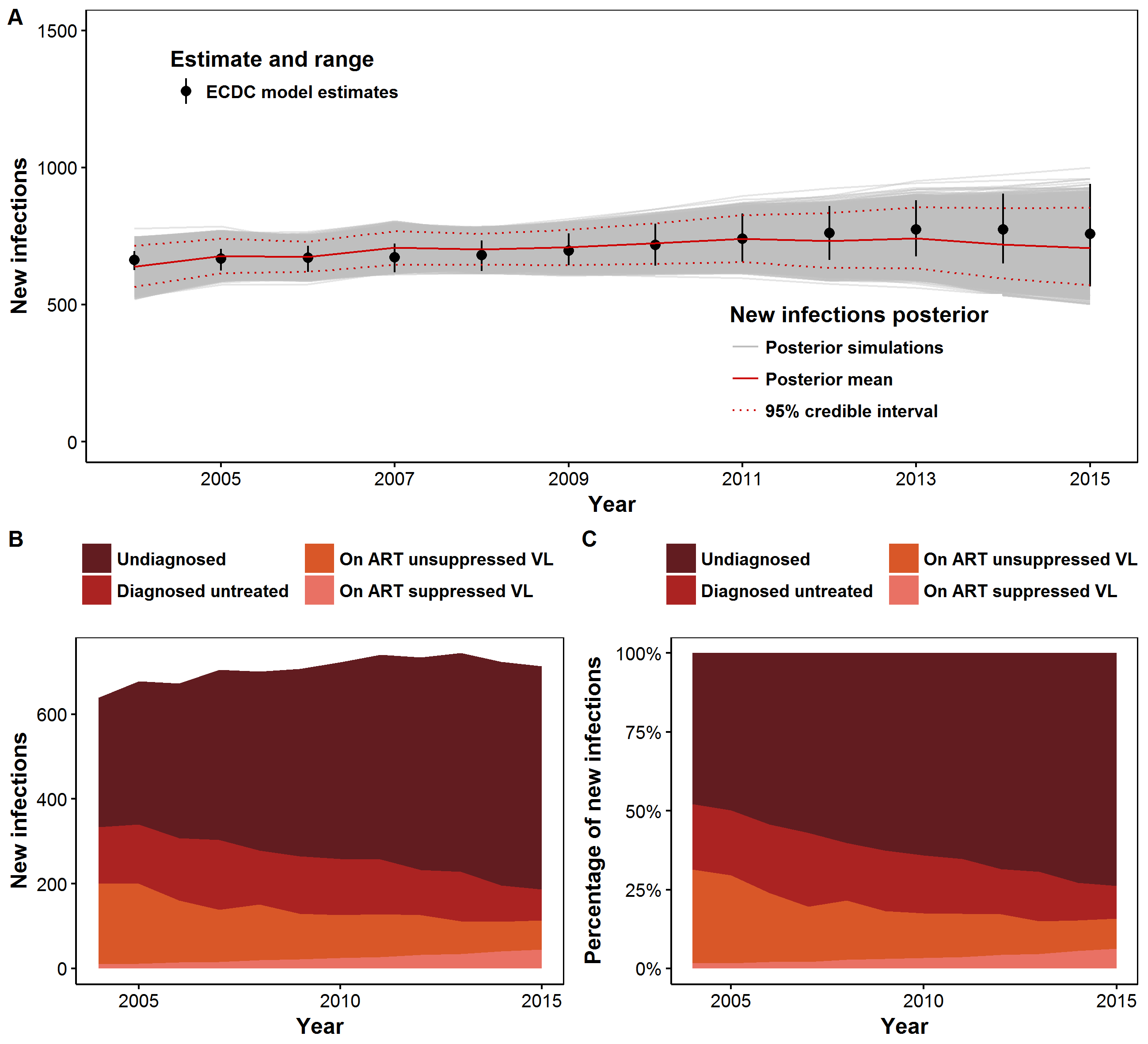


**A)** Estimated new infections for the posterior simulations for the Australian GBM HIV cascade during 2004-2015 with Partner study suppressed transmission coefficient prior and using the 2015 HIV cascade methodology with no range. Each thin grey line is one simulation in the posterior, the thick red line is the posterior mean value at each time point and the dashed red lines are the lower and upper bounds of the 95% credible interval. The black dots and lines show the estimated number new infections and range produced by the ECDC HIV Modelling Tool. Estimated number **B)** and proportion **C)** of overall new infections attributed to each step of the GBM HIV cascade.

**Supplementary Figure S13. The posterior distribution in percentage of new infections attributed to each step of the Australian GBM HIV cascade during 2004-2015 with Partner study suppressed transmission coefficient prior and no range in cascade estimates.**


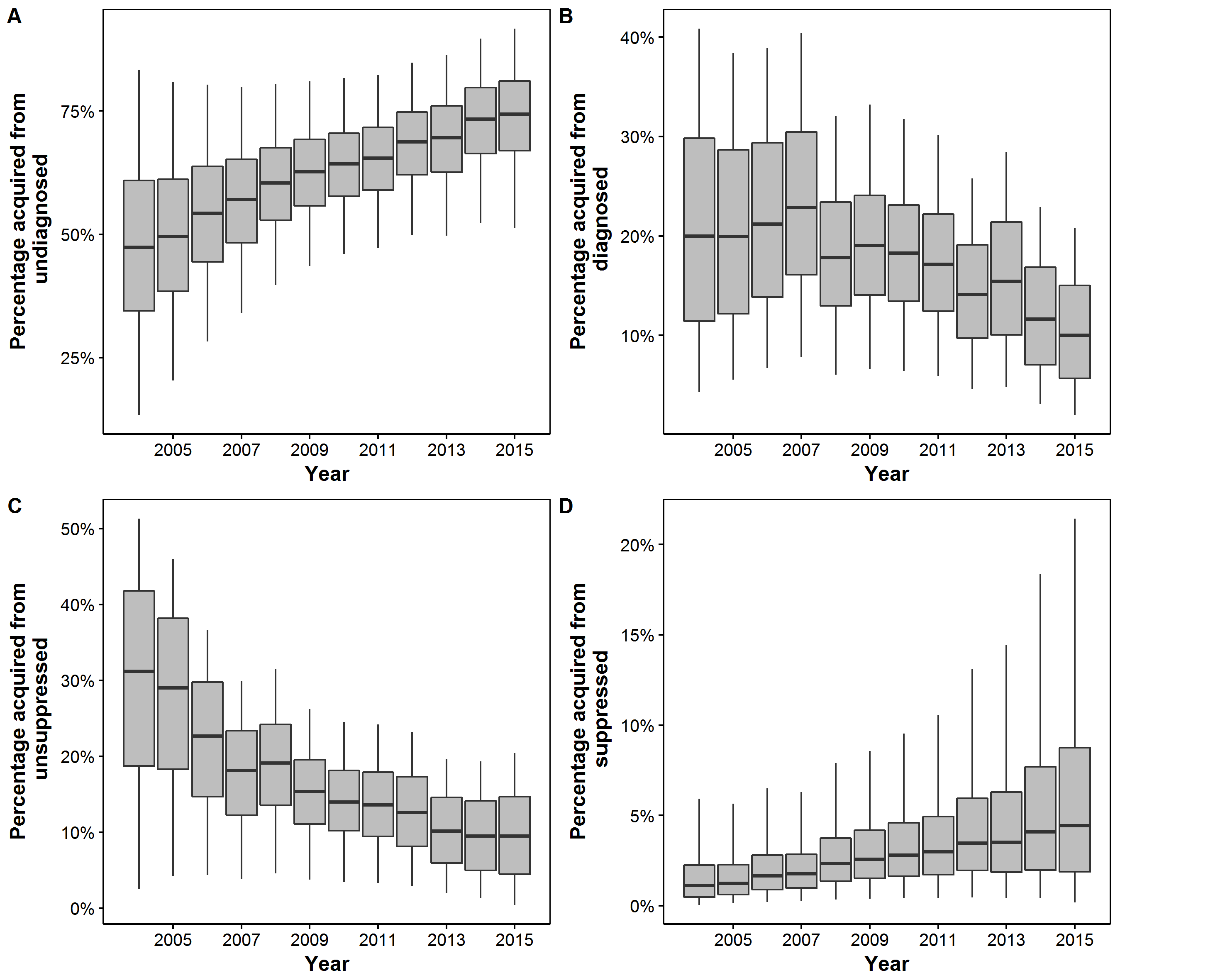


**Supplementary Table S10. New infections attributable to each step of the Australian GBM HIV cascade.**

| **Year** | **Undiagnosed** | | **Diagnosed** | | **Unsuppressed** | | **Suppressed** | | **Total** |
| --- | --- | --- | --- | --- | --- | --- | --- | --- | --- |
|  | Number | Percentage | Number | Percentage | Number | Percentage | Number | Percentage |  |
| 2004 | 306 (84-541) | 47.7% (13.4-83.3%) | 133 (28-256) | 20.9% (4.3-40.9%) | 191 (16-324) | 29.8% (2.5-51.3%) | 10 (0-38) | 1.6% (0-5.9%) | 639 (564-714) |
| 2005 | 338 (137-545) | 49.9% (20.4-81%) | 140 (38-259) | 20.7% (5.5-38.4%) | 189 (29-313) | 27.8% (4.3-46%) | 11 (1-38) | 1.7% (0.1-5.6%) | 677 (615-740) |
| 2006 | 365 (187-549) | 54.2% (28.2-80.4%) | 147 (45-263) | 21.8% (6.7-38.9%) | 147 (30-243) | 21.9% (4.4-36.7%) | 14 (1-44) | 2.1% (0.2-6.5%) | 675 (619-729) |
| 2007 | 401 (237-569) | 56.9% (34-79.9%) | 165 (54-289) | 23.3% (7.8-40.4%) | 124 (28-206) | 17.6% (3.9-29.9%) | 15 (2-44) | 2.1% (0.2-6.3%) | 708 (646-768) |
| 2008 | 423 (274-575) | 60.2% (39.7-80.4%) | 128 (43-221) | 18.2% (6.1-32%) | 132 (32-221) | 18.8% (4.6-31.5%) | 20 (2-55) | 2.8% (0.3-7.9%) | 702 (645-758) |
| 2009 | 443 (303-584) | 62.5% (43.6-81%) | 136 (47-234) | 19.2% (6.6-33.2%) | 108 (27-184) | 15.2% (3.8-26.2%) | 22 (3-60) | 3.1% (0.4-8.6%) | 709 (644-772) |
| 2010 | 464 (326-600) | 64.1% (46-81.7%) | 133 (46-229) | 18.4% (6.4-31.8%) | 102 (25-176) | 14.1% (3.4-24.5%) | 24 (3-68) | 3.4% (0.4-9.5%) | 723 (649-797) |
| 2011 | 483 (340-621) | 65.2% (47.2-82.3%) | 129 (44-221) | 17.4% (5.9-30.1%) | 101 (24-179) | 13.7% (3.3-24.2%) | 27 (3-77) | 3.7% (0.4-10.5%) | 740 (654-826) |
| 2012 | 503 (351-648) | 68.3% (49.9-84.8%) | 106 (34-184) | 14.5% (4.6-25.8%) | 94 (22-170) | 12.8% (3-23.2%) | 32 (3-96) | 4.4% (0.5-13.1%) | 732 (634-834) |
| 2013 | 515 (354-670) | 69.2% (49.8-86.4%) | 118 (36-208) | 15.8% (4.8-28.4%) | 77 (15-143) | 10.4% (2-19.6%) | 34 (3-108) | 4.6% (0.4-14.5%) | 741 (632-855) |
| 2014 | 528 (353-695) | 72.7% (52.3-89.7%) | 86 (23-157) | 12% (3.1-22.9%) | 70 (10-135) | 9.7% (1.4-19.3%) | 41 (3-137) | 5.6% (0.4-18.4%) | 719 (595-851) |
| 2015 | 527 (341-702) | 73.6% (51.3-91.7%) | 73 (15-139) | 10.4% (2-20.8%) | 69 (3-139) | 9.7% (0.4-20.4%) | 45 (1-159) | 6.2% (0.2-21.4%) | 707 (571-853) |

Estimated number (mean and 95% CrI rounded to the nearest whole number) and percentage (mean and 95% credible interval) of new infections attributable to each step of the Australian GBM HIV cascade during 2004-2015 with Partner study suppressed transmission coefficient prior and no range in cascade estimates.

**Supplementary Figure S14.** **Change in the rate of transmission for people living with HIV in each step of the Australian GBM HIV cascade during 2004-2015 with Partner study suppressed transmission coefficient prior and no range in cascade estimates.**


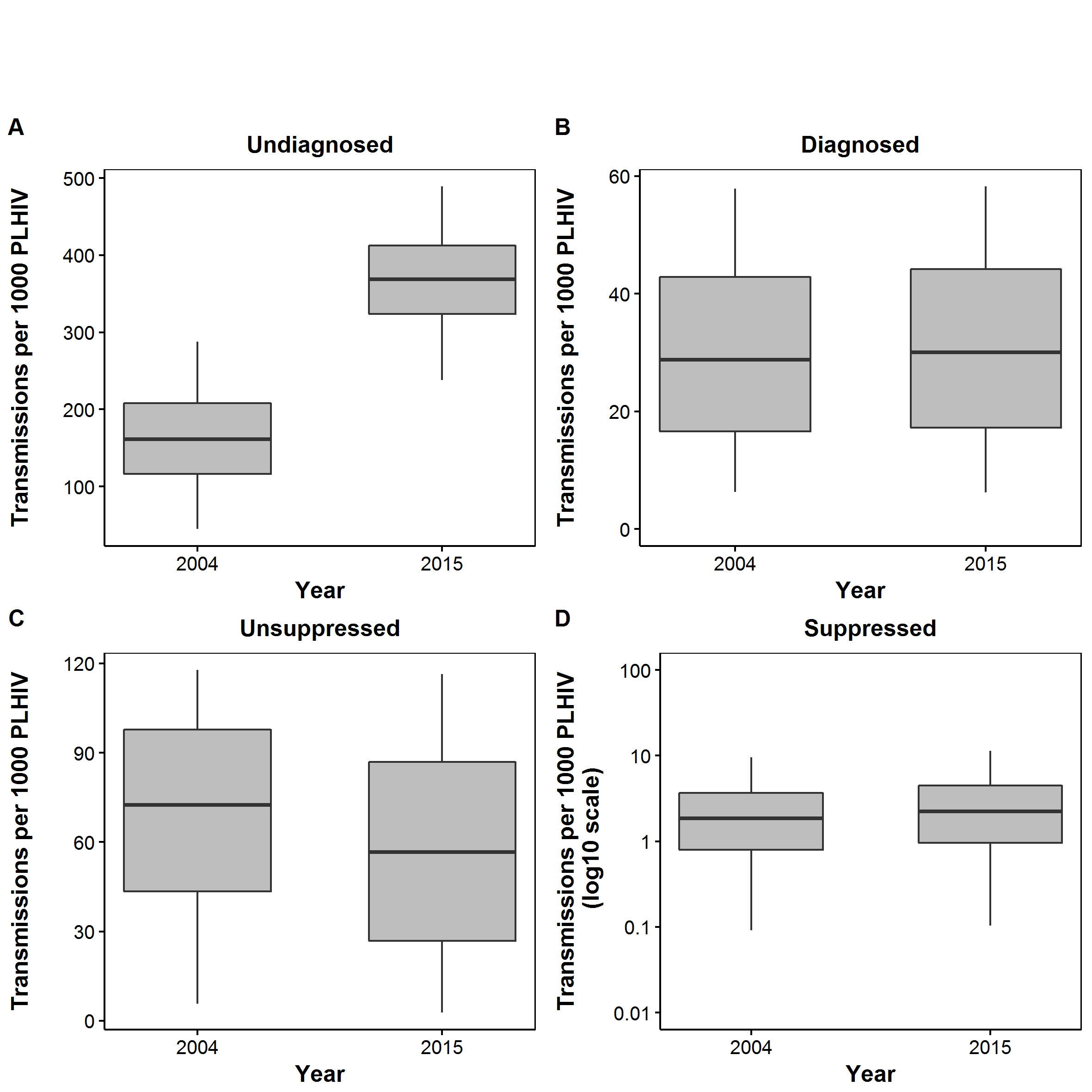


**Supplementary Table S11. Posterior rates of transmission in 2004 and 2015 per 1000 people in each step of the HIV cascade.**

|  | 2004 | | 2015 | |
| --- | --- | --- | --- | --- |
| Step | Value | Relative to diagnosed | Value | Relative to diagnosed |
| Undiagnosed | 160 (45-290) | 9.2 (0.82-37) | 370 (240-490) | 19 (4.6-68) |
| Diagnosed | 30 (6.3-58) | 1 (1-1) | 31 (6.2-58) | 1 (1-1) |
| Unsuppressed | 69 (5.8-120) | 3.4 (0.21-13) | 57 (2.7-120) | 2.8 (0.093-12) |
| Suppressed | 2.6 (0.076-9.6) | 0.13 (0.0024-0.63) | 3.2 (0.093-11) | 0.15 (0.0029-0.73) |

Mean and 95% credible interval of the posterior rates of transmission in 2004 and 2015 per 1000 people in each step of the Australian GBM HIV cascade during 2004-2015 with Partner study suppressed transmission coefficient prior and no range in cascade estimates. Results rounded to two significant figures.

- 1. **Australian GBM HIV cascade During 2004-2014 with Cohen 2011 suppressed transmission coefficient prior and the 2014 HIV cascade methodology**

This section shows the results obtained by our methodology when using the Australian GBM HIV cascade during 2004-2014 (obtained using the 2014 HIV cascade methodology [13]; which assumed a lower overseas migration rate and defines viral suppression as a viral load < 400 copies/ml at last test). The transmission coefficient for the suppressed population had a prior based on the results from the Cohen et al study in 2011. This prior is given by a lognormal distribution with mean 0.04 (95% CI: 0.01-0.27) [12] and describes the relative reduction in the transmission coefficient compared to the diagnosed not on ART population, as shown in Eq. 2.

The first section gives the 2004-2014 Australian GBM HIV cascade estimates based on the alternative methodology and the second section gives the results.

***Estimates for each step of the 2004-2014 cascade and new infections***

**Supplementary Figure S15:** **Estimates for new infections and the Australian GBM HIV cascade during 2004-2014**


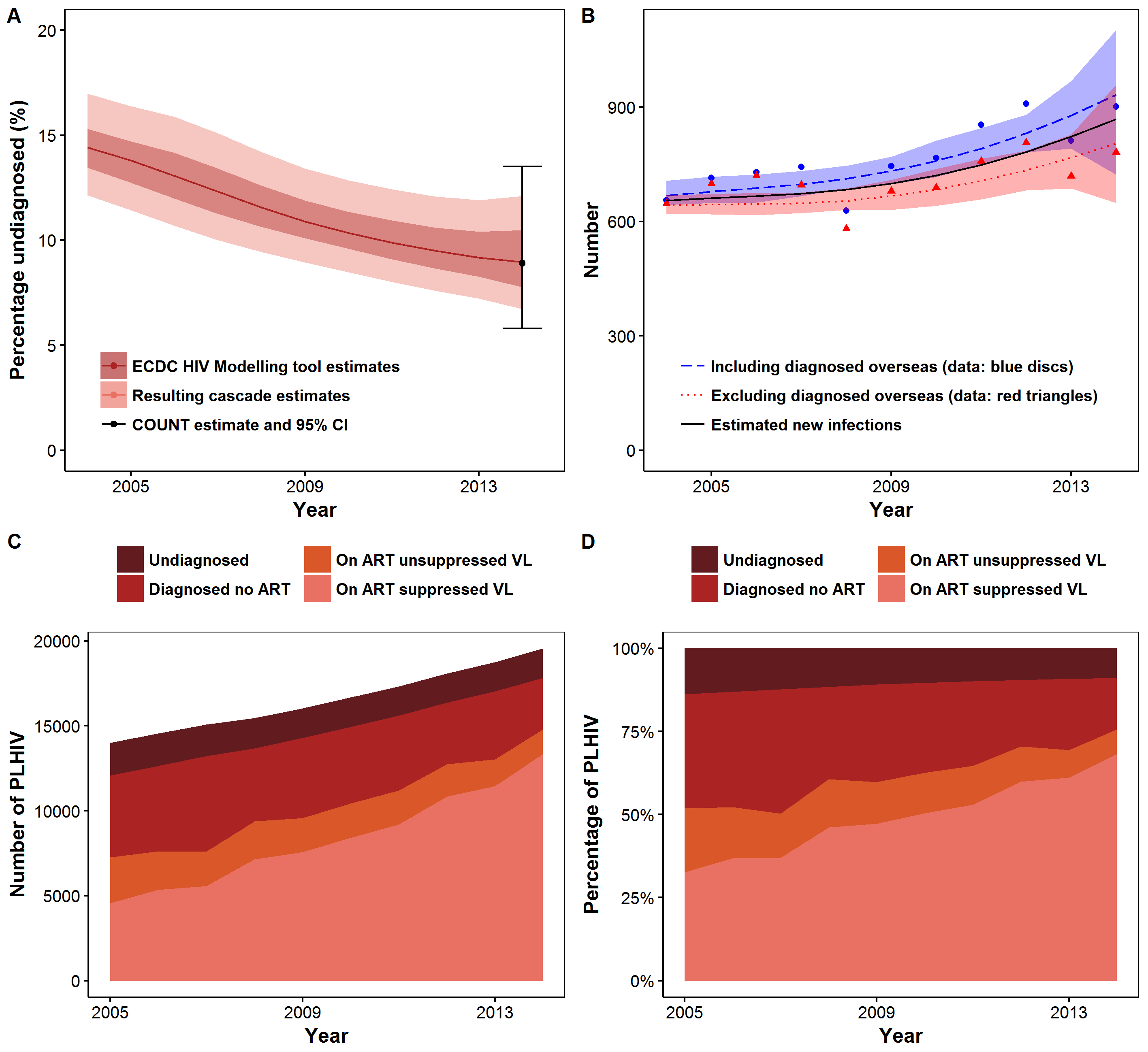


**A)** Estimated percentage of people undiagnosed during 2004-2014 compared to the 2014 COUNT estimate. The red line is the average of the two estimates produced by the two ECDC HIV Modelling Tool scenarios. The dark red band is the range in the percentage undiagnosed from the ECDC tool and the lighter band is the overall range in the percentage undiagnosed once the uncertainty in the number diagnosed is included.. The black disc and error bar show the point estimate and 95% confidence interval for percentage undiagnosed nationally from the COUNT study. **B)** Estimated number of new infections from the ECDC HIV Modelling Tool with people previously diagnosed overseas (blue) and with those people excluded (red). The blue and red points correspond to the respective number of notifications attributed to male-to-male sex. The black line is the best estimate used for the analysis. The HIV cascade for Australian GBM during 2004-2014 with the number **C)** and proportion **D)** of HIV+ GBM who are undiagnosed, diagnosed but not on ART, on ART but with unsuppressed VL, and on ART with suppressed VL.

**Supplementary Figure S16.** **Number of GBM living with HIV in each step of the HIV cascade during 2004-2014 using the 2014 HIV cascade methodology**


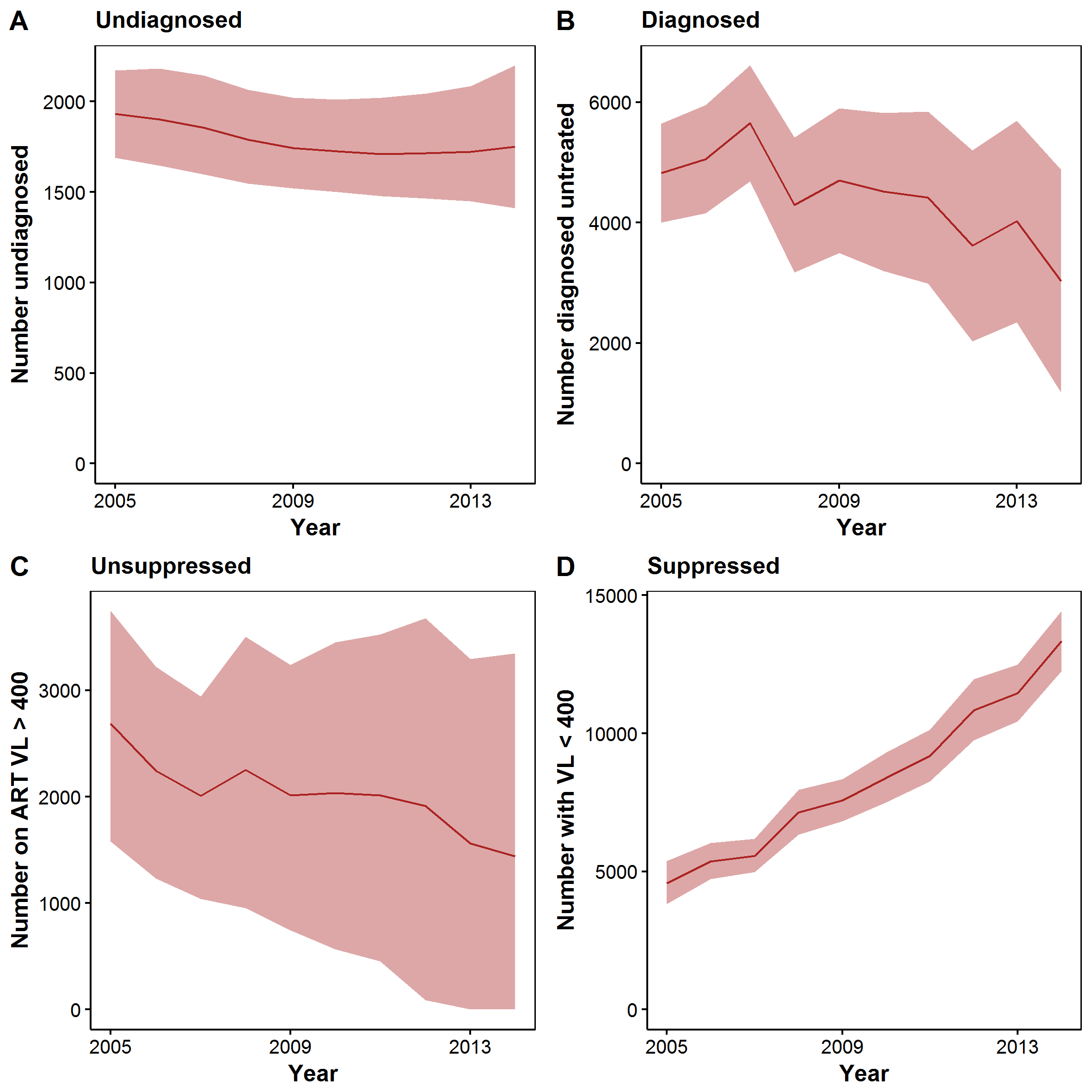


**Supplementary Table S12.** **Australian GBM HIV cascade estimates and ECDC estimates of new infections for 2004-2014.**

|  | Undiagnosed | | Diagnosed | | Unsuppressed | | Suppressed | | New infections |
| --- | --- | --- | --- | --- | --- | --- | --- | --- | --- |
|  | Number | Percentage | Number | Percentage | Number | Percentage | Number | Percentage | Number |
| 2004 | 1930 (1710-2160) | 14.4% (12.1-17%) | 4580 (3830-5330) | 34.1% (27.1-41.9%) | 2760 (1730-3740) | 20.6% (12.3-29.4%) | 4140 (3440-4880) | 30.9% (24.4-38.4%) | 655 (620-707) |
| 2005 | 1930 (1690-2170) | 13.8% (11.4-16.4%) | 4820 (4000-5640) | 34.4% (27.1-42.6%) | 2690 (1580-3750) | 19.2% (10.7-28.3%) | 4570 (3820-5370) | 32.6% (25.9-40.5%) | 662 (619-717) |
| 2006 | 1900 (1650-2180) | 13.1% (10.7-15.9%) | 5050 (4150-5950) | 34.7% (27-43.3%) | 2240 (1230-3220) | 15.4% (8-23.4%) | 5360 (4730-6040) | 36.8% (30.7-43.9%) | 667 (617-723) |
| 2007 | 1860 (1600-2140) | 12.3% (10-15.1%) | 5650 (4680-6610) | 37.4% (29.3-46.5%) | 2010 (1040-2940) | 13.3% (6.5-20.7%) | 5570 (4990-6190) | 36.9% (31.2-43.5%) | 673 (622-733) |
| 2008 | 1790 (1550-2070) | 11.6% (9.4-14.2%) | 4290 (3170-5420) | 27.8% (19.3-37.2%) | 2250 (950-3500) | 14.6% (5.8-24.1%) | 7130 (6330-7970) | 46.1% (38.6-54.7%) | 683 (630-747) |
| 2009 | 1740 (1520-2020) | 10.9% (8.9-13.4%) | 4700 (3490-5900) | 29.3% (20.5-39.1%) | 2010 (740-3240) | 12.6% (4.4-21.5%) | 7570 (6820-8350) | 47.2% (40.1-55.4%) | 699 (630-770) |
| 2010 | 1720 (1500-2010) | 10.3% (8.5-12.8%) | 4510 (3200-5820) | 27.1% (18.1-37.2%) | 2030 (570-3450) | 12.2% (3.2-22%) | 8400 (7510-9320) | 50.4% (42.4-59.5%) | 721 (641-812) |
| 2011 | 1710 (1480-2020) | 9.9% (8-12.4%) | 4420 (2990-5840) | 25.5% (16.2-36%) | 2010 (460-3520) | 11.6% (2.5-21.7%) | 9180 (8260-10130) | 53% (44.8-62.4%) | 749 (658-845) |
| 2012 | 1720 (1460-2050) | 9.5% (7.6-12.1%) | 3620 (2030-5200) | 20% (10.5-30.7%) | 1910 (90-3680) | 10.6% (0.5-21.7%) | 10840 (9760-11960) | 59.9% (50.6-70.6%) | 783 (681-880) |
| 2013 | 1720 (1450-2090) | 9.2% (7.2-11.9%) | 4020 (2340-5690) | 21.4% (11.7-32.5%) | 1560 (0-3290) | 8.3% (0-18.8%) | 11460 (10440-12500) | 61.1% (52-71.3%) | 822 (687-968) |
| 2014 | 1750 (1410-2200) | 9% (6.7-12.1%) | 3030 (1180-4870) | 15.5% (5.6-26.8%) | 1440 (0-3350) | 7.4% (0-18.4%) | 13340 (12260-14430) | 68.2% (58.4-79.3%) | 868 (648-1102) |

Australian GBM HIV cascade estimates and ECDC estimates of new infections for 2004-2014 using the 2014 HIV cascade methodology. These numbers are the input data for the model with the model fitting to the annual number of new infections. All estimates and ranges rounded to the nearest whole number. Percentage for each cascade step is the percentage of all people living with HIV.

***Results***

**Supplementary Figure S17. Estimated number and percentage of new infections attributed to each step of the Australian GBM HIV cascade.**


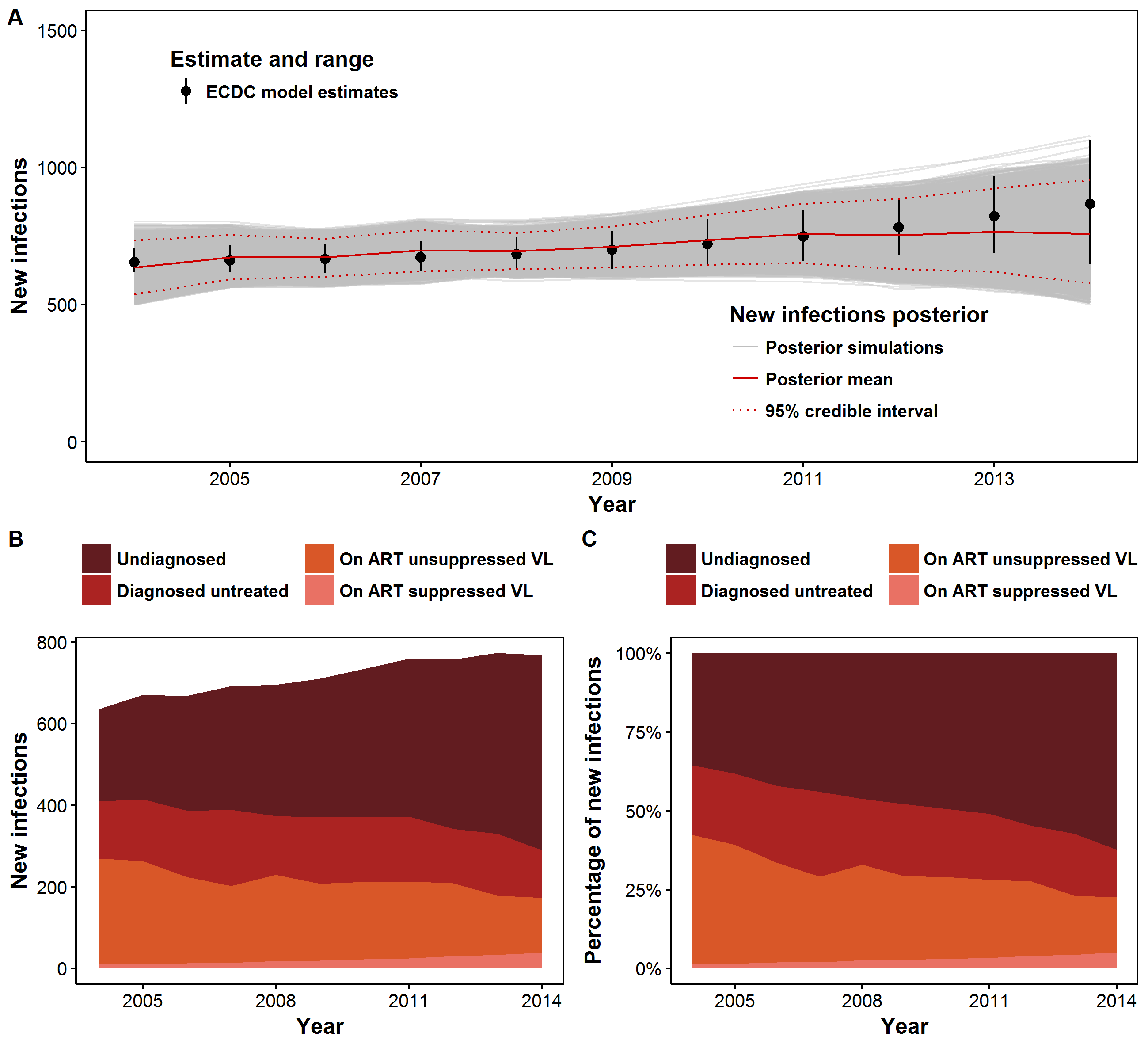


**A)** Estimated new infections for the posterior simulations for the Australian GBM HIV cascade during 2004-2014 with Cohen 2011 suppressed transmission coefficient prior and using the 2014 HIV cascade methodology. Each thin grey line is one simulation in the posterior, the thick red line is the posterior mean value at each time point and the dashed red lines are the lower and upper bounds of the 95% credible interval. The black dots and lines show the estimated number new infections and range produced by the ECDC HIV Modelling Tool. Estimated number **B)** and proportion **C)** of overall new infections attributed to each step of the GBM HIV cascade.

**Supplementary Figure S18.** **The posterior distribution in percentage of new infections attributed to each step of the Australian GBM HIV cascade during 2004-2014 with Cohen 2011 suppressed transmission coefficient prior and using the 2014 HIV cascade methodology.**


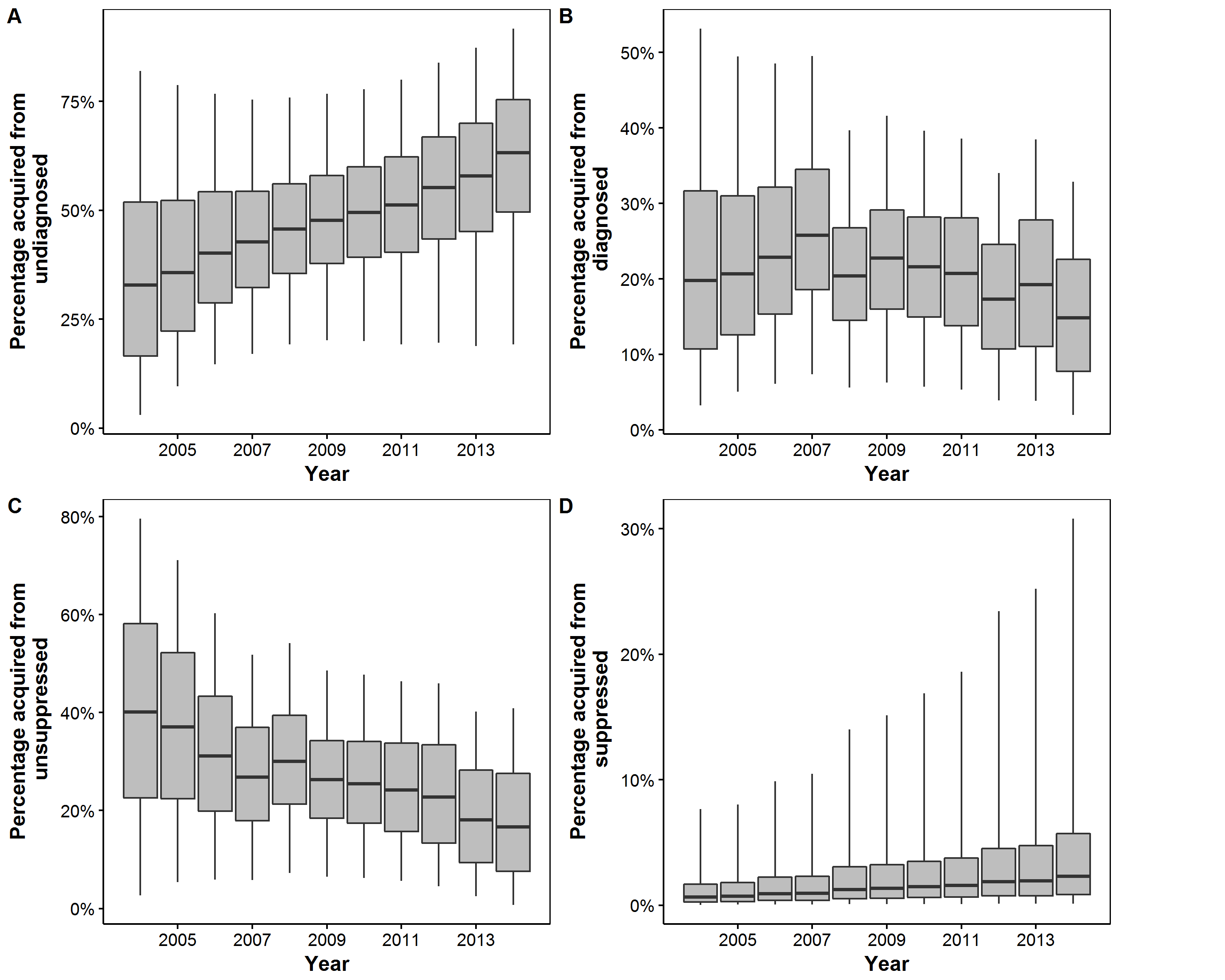


**Supplementary Table S13. New infections attributable to each step of the Australian GBM HIV cascade during 2004-2014.**

| **Year** | **Undiagnosed** | | **Diagnosed** | | **Unsuppressed** | | **Suppressed** | | **Total** |
| --- | --- | --- | --- | --- | --- | --- | --- | --- | --- |
|  | Number | Percentage | Number | Percentage | Number | Percentage | Number | Percentage |  |
| 2004 | 226 (19-522) | 35.7% (3.1-82%) | 140 (21-327) | 22.3% (3.2-53.2%) | 260 (17-524) | 40.6% (2.7-79.6%) | 9 (0-48) | 1.5% (0-7.7%) | 636 (537-735) |
| 2005 | 256 (65-525) | 38.3% (9.6-78.7%) | 152 (34-328) | 22.7% (5-49.5%) | 253 (36-483) | 37.5% (5.4-71.2%) | 11 (0-54) | 1.6% (0.1-8%) | 673 (592-752) |
| 2006 | 282 (96-526) | 42% (14.7-76.7%) | 163 (41-331) | 24.3% (6.1-48.5%) | 211 (40-388) | 31.8% (5.9-60.3%) | 13 (0-66) | 1.9% (0.1-9.9%) | 673 (602-740) |
| 2007 | 304 (116-527) | 43.8% (17.1-75.4%) | 186 (49-361) | 26.7% (7.4-49.5%) | 189 (41-341) | 27.5% (5.8-51.8%) | 14 (0-72) | 2% (0.1-10.5%) | 699 (621-772) |
| 2008 | 321 (132-532) | 46.1% (19.2-75.9%) | 145 (40-271) | 20.9% (5.6-39.7%) | 211 (50-381) | 30.3% (7.2-54.2%) | 18 (1-98) | 2.6% (0.1-14%) | 696 (629-762) |
| 2009 | 340 (142-546) | 47.8% (20.1-76.8%) | 162 (44-297) | 22.8% (6.2-41.6%) | 188 (46-343) | 26.6% (6.5-48.6%) | 20 (1-104) | 2.8% (0.1-15.2%) | 712 (636-786) |
| 2010 | 363 (146-574) | 49.4% (20-77.8%) | 159 (41-288) | 21.7% (5.7-39.6%) | 190 (45-350) | 25.9% (6.3-47.8%) | 22 (1-124) | 3% (0.1-16.9%) | 735 (645-825) |
| 2011 | 387 (146-608) | 50.9% (19.2-80%) | 159 (40-288) | 21% (5.3-38.6%) | 188 (42-353) | 24.8% (5.6-46.4%) | 25 (1-140) | 3.3% (0.1-18.6%) | 758 (651-868) |
| 2012 | 415 (145-664) | 54.6% (19.6-83.9%) | 133 (30-243) | 17.8% (3.9-34%) | 178 (34-343) | 23.6% (4.5-46%) | 30 (1-177) | 4% (0.1-23.4%) | 753 (628-885) |
| 2013 | 443 (137-718) | 56.9% (18.9-87.3%) | 151 (29-285) | 19.7% (3.8-38.5%) | 146 (20-291) | 19.2% (2.5-40.2%) | 33 (1-195) | 4.2% (0.1-25.2%) | 766 (619-924) |
| 2014 | 478 (130-793) | 61.3% (19.3-91.7%) | 116 (16-228) | 15.5% (2-32.8%) | 134 (6-282) | 18% (0.7-40.9%) | 39 (1-232) | 5.1% (0.1-30.8%) | 757 (577-954) |

Estimated number (mean and 95% CrI rounded to the nearest whole number) and percentage (mean and 95% credible interval) of new infections attributable to each step of the Australian GBM HIV cascade during 2004-2014 with Cohen 2011 suppressed transmission coefficient prior and using the 2014 HIV cascade methodology.

**Supplementary Figure S19.** **Change in the rate of transmission for people living with HIV in each step of the of the Australian GBM HIV cascade during 2004-2014 with Cohen 2011 suppressed transmission coefficient prior and using the 2014 HIV cascade methodology.**


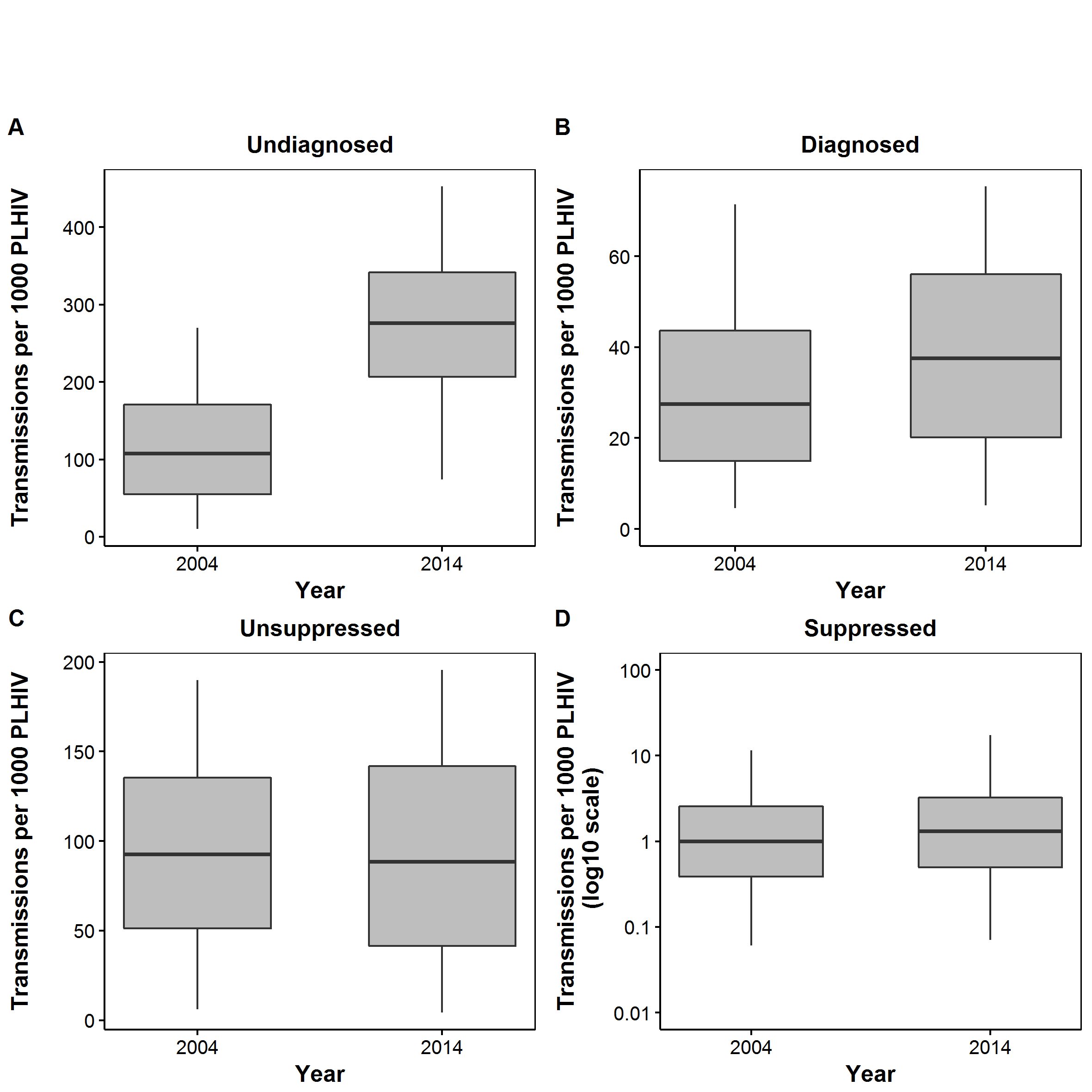


**Supplementary Table S14.** **Posterior rates of transmission in 2004 and 2014 per 1000 people in each step of the HIV cascade.**

|  | 2004 | | 2014 | |
| --- | --- | --- | --- | --- |
| Step | Value | Relative to diagnosed | Value | Relative to diagnosed |
| Undiagnosed | 120 (10-270) | 7.8 (0.23-43) | 270 (74-450) | 14 (1.2-70) |
| Diagnosed | 31 (4.6-71) | 1 (1-1) | 38 (5.2-75) | 1 (1-1) |
| Unsuppressed | 94 (6.1-190) | 5.6 (0.17-27) | 93 (4.3-200) | 4.2 (0.1-22) |
| Suppressed | 2.2 (0.06-12) | 0.076 (0.004-0.39) | 2.9 (0.07-17) | 0.076 (0.004-0.39) |

Mean and 95% credible interval of the posterior rates of transmission in 2004 and 2014 per 1000 people in each step of the Australian GBM HIV cascade during 2004-2014 with Cohen 2011 suppressed transmission coefficient prior and using the 2014 HIV cascade methodology. Results rounded to two significant figures.

1. **References**

1. The Kirby Institute. HIV, viral hepatitis and sexually transmissible infections in Australia Annual Surveillance Report 2016. The Kirby Institute, UNSW Sydney, Sydney, Australia, 2016.

2. Gray RT, leftygray/Cascade_calculations: Version used for the 2017 Annual Surveillance Report (version v3.0_2017_ASR). Zenodo; 2017 [http://doi.org/10.5281/zenodo.998280]. Also available from: https://github.com/leftygray/Cascade_calculations

3. van Sighem A, Nakagawa F, De Angelis D, Quinten C, Bezemer D, de Coul EO, et al. Estimating HIV Incidence, Time to Diagnosis, and the Undiagnosed HIV Epidemic Using Routine Surveillance Data: Epidemiology. 2015 Sep;26:653–60.

4. ECDC HIV modelling tool [software application]. Version 1.2.2. European Centre for Disease Prevention and Control, Stockholm, Sweden, 2016. Available from: http://ecdc.europa.eu/en/healthtopics/aids/Pages/hiv-modelling-tool.aspx

5. Gray RT. leftygray/Cascade_Incidence: Version corresponding to final manuscript. (Version v1.1-final). Zenodo. 2017. [http://doi.org/10.5281/zenodo.1117423]. Also available from: https://github.com/leftygray/Cascade_Incidence

6. Mao JL, Adam P, Treloar C, and de Wit J (Eds). HIV/AIDS, hepatitis and sexually transmissible infections in Australia: annual report of trends in behaviour 2016. Centre for Social Research in Health, UNSW Sydney, Sydney, Australia, 2016.

7. Holt M, Lea T, Mao L, Zablotska I, Lee E, Hull P, et al. Adapting behavioural surveillance to antiretroviral-based HIV prevention: reviewing and anticipating trends in the Australian Gay Community Periodic Surveys. Sex Health. 2017;14:72-79.

8. Hallett TB, Gregson S, Mugurungi O, Gonese E, Garnett GP. Assessing evidence for behaviour change affecting the course of HIV epidemics: a new mathematical modelling approach and application to data from Zimbabwe. Epidemics. 2009;1:108–17.

9. Alkema L, Raftery a E, Brown T. Bayesian melding for estimating uncertainty in national HIV prevalence estimates. Sex Transm Infect. 2008 Aug;84 Suppl 1:i11–i16.

10. Poole D, Raftery AE. Inference for Deterministic Simulation Models: The Bayesian Melding Approach. J Am Stat Assoc. 2000 Dec;95:1244.

11. Rodger AJ, Cambiano V, Bruun T, Vernazza P, Collins S, Van Lunzen J, et al. Sexual activity without condoms and risk of HIV transmission in serodifferent couples when the HIV-positive partner is using suppressive antiretroviral therapy. Jama. 2016;316:171–181.

12. Cohen MS, Chen YQ, McCauley M, Gamble T, Hosseinipour MC, Kumarasamy N, et al. Prevention of HIV-1 infection with early antiretroviral therapy. N Engl J Med. 2011;365:493–505.

13. The Kirby Institute. HIV, viral hepatitis and sexually transmissible infections in Australia Annual Surveillance Report 2015. The Kirby Institute, UNSW Sydney, Sydney, Australia, 2015.
